# Respiration climacteric in tomato fruits elucidated by constraint‐based modelling

**DOI:** 10.1111/nph.14301

**Published:** 2016-11-15

**Authors:** Sophie Colombié, Bertrand Beauvoit, Christine Nazaret, Camille Bénard, Gilles Vercambre, Sophie Le Gall, Benoit Biais, Cécile Cabasson, Mickaël Maucourt, Stéphane Bernillon, Annick Moing, Martine Dieuaide‐Noubhani, Jean‐Pierre Mazat, Yves Gibon

**Affiliations:** ^1^ UMR 1332 Biologie du Fruit et Pathologie INRA Villenave d'Ornon F‐33883 France; ^2^ Université de Bordeaux 146 rue Léo‐Saignat Bordeaux Cedex F‐33076 France; ^3^ ENSTBB‐Institut Polytechnique de Bordeaux Institut de Mathématiques de Bordeaux 351 Cours de la Liberation Talence 33400 France; ^4^ UR 1115 Plantes et Systèmes de culture Horticoles INRA Avignon Cedex 9 F84914 France; ^5^ UR 1268 Biopolymères, Interactions, Assemblages INRA Nantes F‐44316 France; ^6^ IBGC‐CNRS 1 rue Camille Saint‐Saëns Bordeaux Cedex F‐33077 France

**Keywords:** carbon allocation, central metabolism, constraint‐based model, environmental stress, flux balance analysis (FBA), metabolic flux modelling, respiration climacteric, tomato fruit development

## Abstract

- Tomato is a model organism to study the development of fleshy fruit including ripening initiation. Unfortunately, few studies deal with the brief phase of accelerated ripening associated with the respiration climacteric because of practical problems involved in measuring fruit respiration.
- Because constraint‐based modelling allows predicting accurate metabolic fluxes, we investigated the respiration and energy dissipation of fruit pericarp at the breaker stage using a detailed stoichiometric model of the respiratory pathway, including alternative oxidase and uncoupling proteins. Assuming steady‐state, a metabolic dataset was transformed into constraints to solve the model on a daily basis throughout tomato fruit development.
- We detected a peak of CO_2_ released and an excess of energy dissipated at 40 d post anthesis (DPA) just before the onset of ripening coinciding with the respiration climacteric. We demonstrated the unbalanced carbon allocation with the sharp slowdown of accumulation (for syntheses and storage) and the beginning of the degradation of starch and cell wall polysaccharides. Experiments with fruits harvested from plants cultivated under stress conditions confirmed the concept.
- We conclude that modelling with an accurate metabolic dataset is an efficient tool to bypass the difficulty of measuring fruit respiration and to elucidate the underlying mechanisms of ripening.

## Introduction

Due to its agronomic importance, tomato (*Solanum lycopersicum*) has been studied intensively with respect to genetics, physiology and biochemistry, and became an important model for fruit development. For climacteric fruit species, such as tomato, fruit maturation is characterized not only by a metabolic shift from normal development conditions toward the fully mature state, but also by a brief phase of accelerated ripening with a sharp increase in respiration (Andrews, 1995). Ethylene in concert with other plant hormones is known to regulate ripening, and transcription factors play a central role in the high‐level regulatory network controlling fruit maturation and ripening (Seymour *et al*., 2013). But whereas ripening and its regulation have been studied extensively using molecular approaches, less is known about physiological changes occurring during ripening such as the respiratory climacteric.

It has been reported that for climacteric fruits, such as tomato, mango and kiwifruit, the conversion of starch into soluble sugars is one of the most important events during ripening. These fruits store imported photosynthates in the form of starch in amyloplasts and, as ripening proceeds, this carbon is exported to the cytosol and is thought to be converted into sugars and CO_2_ (Moing *et al*., 2001; Han & Kawabata, 2002). The increase in respiration is concomitant with increased ATP levels and perhaps with an increase in energy charge in the cells. Because ATP concentration increases through the climacteric period, the increase in respiration would provide a supply of chemical energy in excess of the demands of the tissue (Brady, 1987). But as mentioned by Sweetlove *et al*. (2013), the term respiration has several meanings depending on the scale at which the process is considered and also because of the multifaceted nature of oxidative metabolism in plants. For instance, at the physiological scale, respiration is defined as the release of CO_2_, whereas at the biochemical level, the focus tends to be an oxidative metabolism.

From a biochemical point of view, plant respiration has evolved as a complex metabolic network with unique characteristics. Plants have the option of employing alternative enzymes that bypass several of the conventional steps in the mitochondrial oxidative phosphorylation (Oxphos) system (Plaxton & Podesta, 2006). Three main proteins modulating the overall yield of Oxphos have been described: type II NAD(P)H dehydrogenases that bypasses complex I and do not pump proton; the alternative oxidases (AOXs) (EC 1.10.3.11) non‐proton‐motive terminal oxidases that bypass complexes III and IV, and significantly decrease the proton pumping efficiency of the electron transport chain (miETC, Rasmusson *et al*., 2008); and the plant uncoupling mitochondrial proteins (UCPs) that increase the proton diffusion across the inner mitochondrial membrane and circumvent the ATP synthase complex. In these alternative pathways, as several proton‐pumping steps are bypassed, the ATP yield is reduced and energy is dissipated. For instance, AOXs theoretically are able to decrease the respiratory ATP output by *c*. 60% (Rasmusson *et al*., 2008). AOXs and UCPs have developmental stage‐specific regulation in plants and each isoform of AOXs and UCPs may have a different physiological role in the process of plant metabolism (Zhu *et al*., 2011). For instance under stress conditions, AOXs are involved in the regulation of reactive oxygen species (ROS) and participate in the signalling system of plants under pathogen attack (Affourtit *et al*., 2001). Similarly, UCPs also have been suggested to prevent generation of ROS by the respiratory chain during fruit ripening (Almeida *et al*., 1999). Expression analyses and functional studies on the plant UCPs suggest that UCPs regulate energy metabolism in the cellular responses to stress through regulation of the proton electrochemical potential difference (delta μH^+^), and a distinctive feature of UCPs is their activation by free fatty acids (Vercesi *et al*., 2006). Recently, AOXs have been associated with fruit ripening of climacteric fruits such as mangoes and tomatoes (Considine *et al*., 2001; Xu *et al*., 2012). Xu *et al*. (2012) confirmed the role of AOXs in tomato fruit ripening with reduced AOX activity in transgenic tomato plants exhibiting retarded ripening and reduced respiration with no apparent respiratory climacteric. Qualitatively, all of these observations indicate physiological circumstances where mitochondrial respiration exerts a certain degree of control on plant metabolism and fruit ripening (Perotti *et al*., 2014).

More theoretically, mathematical modelling of metabolism is particularly promising as it offers systems biology approaches enabling the characterization of the behaviour of complex metabolic systems. Modelling fluxes through the metabolic pathways, which supply cofactors and constituents for biomass synthesis, provides an active depiction of metabolic phenotypes. Constraint‐based modelling such as flux balance analysis (FBA) allows the prediction of metabolic steady‐state fluxes by applying mass balance constraints to a stoichiometric model. Although metabolic modelling in plant cells is not as advanced as in other organisms such as microorganisms and mammalian cells (Oberhardt *et al*., 2009), considerable progress has been made in recent years in the study of metabolism in plants (for more details, see the review of Shi & Schwender, 2016). In tomato, a genome‐scale metabolic model of tomato recently has been reconstructed and used to simulate tomato leaf metabolism (Yuan *et al*., 2016), and a constraint‐based model has been used to calculate relevant fluxes throughout tomato fruit development (Colombié *et al*., 2015). Glycolysis, tricarboxylic acid (TCA) cycle and miETC are central features of carbon metabolism and bioenergetics in aerobic organisms in which respiration is connected with sugar supply. One major interest with constraint‐based modelling is the possibility to study the energy metabolism under conditions where both redox and energy cofactors are balanced. To achieve that possibility, the demand on the energy budget due to cell maintenance for protein turnover, ionic gradients and substrate cycles has to be considered (Cannell & Thornley, 2000). Although respiration is essential for growth and maintenance of plant tissues, this process is poorly investigated and integrated in plant or ecosystem models. Cheung *et al*. (2013) claimed that accounting for maintenance costs in flux balance analysis improves the prediction of plant cell metabolic phenotypes under stress conditions of heterotrophic *Arabidopsis* cells in culture. To sum up, Sweetlove *et al*. (2013) pointed out that with a complete and correct list of reactions, and the accuracy of the experimentally measured constraints, FBA can predict fluxes of each reaction in the network including respiration – that is, fluxes that produce CO_2_. Moreover, if there is information on how the constraints respond to environmental changes, such as the effect of water stress on biomass composition, then FBA can be useful to predict relationships between the environment and net CO_2_ evolution rates.

The lack of technically reliable devices applicable to attached fruits has led to only few studies dealing with fruit respiration from a physiological prospect (Atkin, 2011). The aim of the present study was to focus on tomato fruit development using a stoichiometric model to accurately describe metabolic fluxes and especially respiration. With a high temporal model resolution, that is, on a daily basis after anthesis, the simulations of the model revealed an unexpected peak of CO_2_ released flux and energy dissipation which occurs just before maturation and coincides with the respiration climacteric. To elucidate the peak, we changed the metabolic constraints arbitrarily and used contrasting datasets acquired with plants grown under stress conditions (water limitation and shading) that modified the fruit carbon content. Metabolic modelling appears to be a way to fill the gap in our knowledge about fruit respiration because it gets around the difficulty of measuring gas exchange of fruits attached to the plant.

## Materials and Methods

### Plant material and growth conditions

Experiments were performed with the *Solanum lycopersicum* L. Moneymaker cultivar as described in Biais *et al*. (2014). Briefly, tomato plants were grown in a glasshouse in South–west France (44°23ʹ56ʹʹN, 0°35ʹ25ʹʹE) according to usual production practices from June to October and flower anthesis was recorded. The irradiance outside the glasshouse also was recorded. The nutrient solutions used were adapted to plant growth and the water supply was adjusted to the climate using a drip irrigation system to maintain either 20–30% drainage for control and shaded or 5% drainage for water‐stressed conditions (pH adjusted to 5.9, electrical conductivity to 2.2 mS cm^−1^). Photosynthetically active radiation (PAR) and air temperature were measured using a PAR Quantum Sensor (LI‐190; Li‐Cor, Lincoln, NE, USA) and resistance temperature detectors (PT‐100) located in a ventilated shelter at the top of the canopy, respectively. The temperature of eight growing fruits per condition (control, water stressed and shaded) was measured using thermocouples (type K) positioned in the pericarp on the bottom of the first fruit of the fifth truss during three successive periods: from 12 to 26 August, from 3 to 21 September, and from 22 September to 3 October.

Fruits at nine developmental stages (8, 15, 21, 28, 34, 42‐mature green, 49‐turning, 50‐orange and 53‐red ripe d post anthesis, DPA) were harvested on three different trusses (trusses 5, 6 and 7). For each sample, three biological replicates were prepared with a minimum of four fruits per replication. Samples were prepared by first removing the seeds, jelly and placenta, and then cutting the fruit pericarp into small pieces and immediately freezing them in liquid nitrogen. Samples were ground and stored at −80°C until analysis.

### Metabolite contents and biomass composition

Experimental procedures and data on the three major soluble sugars (sucrose, glucose and fructose), total and individual amino acids, organic acids (malate and citrate), starch and protein content are reported in Biais *et al*. (2014). Total cell wall polysaccharides were analysed on dry samples at the BIBS platform (Nantes, France), lipids and total DNA content were analysed on dry samples using the protocols detailed in Colombié *et al*. (2015). Given the high reproducibility of the biochemical composition of fruits irrespective of the truss (Biais *et al*., 2014), the analyses performed on the three trusses were averaged at each developmental stage.

Fumarate, free galactose and galacturonate were quantified using ^1^H‐NMR analyses of polar extracts issued from 20 mg of lyophilized powder as described previously (Bénard *et al*., 2015). Metabolite concentrations were calculated using selected peak areas and calibration curve data.

The total carbon and nitrogen contents were determined in dry samples according to the Dumas method with an elementary auto analyzer (Flash EA 1112 series; Thermo Fisher Scientific, Courtaboeuf, France).

### Fitting and modelling

Concentrations of accumulated metabolites and biomass components were fitted in order to calculate, by derivation, the corresponding fluxes used as boundaries in the flux balance model. To this end, a regression method was used to evaluate the derivative as precisely as possible at each stage of development. The best fit in the least‐squares sense which minimizes the sum of squared residuals, that is, the difference between observed and fitted values, was provided by a polynomial fitting method (details in Colombié *et al*., 2015). As the data became more scattered in older fruits, data were first log‐transformed to minimize heteroscedasticity in the residual terms. Once the transformation and the fit were applied, the values of the derivative (i.e. the rates of accumulation and degradation of metabolites and biomass components) were used as constraints to the corresponding fluxes. Because fewer experimental data were collected under shaded conditions, a lower polynomial degree than for control (generally two instead of three) was chosen as it was statistically better. Biological variability was considered by taking the 95% interval confidence of the fit calculated by the regression method. Mathematical problems were implemented using Matlab (Mathworks R2012b, Natick, MA, USA) and the optimization toolbox, solver *quadprod* with interior‐point‐convex algorithm for the minimization.

## Results

### Stoichiometric model of tomato fruit cells

The flux‐balance model used in this study, which is based on previous work (Beurton‐Aimar *et al*., 2011; Colombié *et al*., 2015), is a medium‐scale knowledge‐based model describing central metabolism of heterotrophic plant cells. It simulates the functioning of the tomato fruit pericarp during fruit development, that is, with breakdown and transformation of nutriments to produce energy and metabolic precursors of biomass components (sugars, organic acids, amino acids, proteins, cell wall, etc.). The corresponding network is available (Supporting Information Notes S1) with a scheme and the list of reactions (Table S1). The main biosynthetic processes were described with overall reactions for protein synthesis (*Vprotein*), fatty acid synthesis (diacyl glycerol, *Vdag*) and nucleotide synthesis (DNA and RNA, *Vnucleotides*). The other accumulated compounds were described as simple vacuolar storage: (1) organic acids, that is, malate (*Vac‐mal*) and citrate (*Vac‐cit*); (2) soluble sugars, that is, glucose (*Vac‐glc*), fructose (*Vac‐fru*) and sucrose (*Vac‐suc*); and (3) four groups of free amino acids, that is, glutamate (*Vac‐Glu*), aspartate (*Vac‐Asp*), alanine (*Vac‐Ala*) and serine (*Vac‐Ser*). Compared with the previous version (Colombié *et al*., 2015), one reaction was added for cell wall degradation (*Vcwd*) in addition to cell wall synthesis (*Vcws*), in order to split synthesis and degradation similarly to starch (*Vsd* and *Vss* for degradation and synthesis, respectively). These 16 fluxes linked to accumulated metabolites and biomass components were defined as outfluxes and were evaluated by fitting the experimental concentrations as described in Colombié *et al*. (2015).

Because biosynthesis of the main biomass components (cell wall, proteins, lipids, starch) requires both ATP and NAD(P)H, energy costs were explicitly taken into account. The cofactors NADP/NADPH were linked to biomass production, and the cofactors NAD/NADH and FAD/FADH were linked to ATP synthesis via a cascade of reactions associated to the miETC described by the ‘standard’ pathway also termed the ‘cytochrome pathway’. The first reactions regenerating the cofactors (*Vrcc1* and *Vrcc2* for the respiratory chain complexes one and two associated, respectively, with NADH and FADH2) involved ubiquinone (UQ/UQH2) which was reoxidized mainly by the cytochrome pathway (*Vrcc34*) associated with proton transfer from the matrix (Hi) to the intermembrane space (He). Back‐diffusion of protons to the matrix is coupled to the synthesis of ATP from ADP and Pi, through the activity of ATP‐synthase plus ATP/ADP and Pi carriers (*Vatpsynthase*). In our model, bypasses of the classical pathway were set by adding AOXs (*Vaox*) which directly couples the oxidation of ubiquinol with the O_2_ reduction and UCPs (*Vucp*) which dissipates protons similarly as proton leak. The non‐proton‐pumping NADH dehydrogenases were also taken into account for energy dissipation. Based on differences in capacity and affinity of complex I and alternative NADH dehydrogenases (Moller, 2001, for review), an equal contribution of these enzymes was assumed in the model thus setting a proton stoichiometry value of 2 in *Vrcc1*. The portion of synthesized ATP that was not used for growth has been balanced in the model as an ATP hydrolysing reaction (*Vnga‐ATPm*) that would represent at least a part of cellular maintenance. Thus, in the model respiratory metabolism was described by a set of seven equations:

*Vrcc1*: NADH + UQ + 2 Hi ⇒ NAD + UQH2 + 2 He
*Vrcc2*: FADH2 + UQ ⇒ FAD + UQH2
*Vrcc34*: 6 Hi + UQH2 + 1/2 O_2_ ⇒ 6 He + UQ + H_2_O
*Vatpsynthase*: 4 He + ADP + Pi ⇒ 4 Hi + ATP
*Vaox*: UQH2 ⇒ UQ
*Vucp*: He ⇒ Hi
*Vnga‐atpm*: ATP ⇒ ADP.

Recycling of the AMP produced from biosynthesis by adenylate kinase was described by *Vadk*. Finally, all cofactors were defined as ‘internal metabolites’, which means that they were balanced. In consequence the metabolic network was not only constrained by carbon and nitrogen balance but also by the redox and energy status.

The whole fruit was considered as the modelled system and all of the fluxes were first calculated on a fruit basis (in mmol per fruit d^−1^) in order to take into account fruit expansion. Subsequently, to enable comparisons between, for example, environmental conditions, all fluxes were converted and expressed on a gram basis, by using the fruit growth curve (Table S2).

### Constraints limiting the flux space and resolution of fluxes

Four types of constraints were applied in order to limit the flux solution space.

First we assumed steady‐state at each stage to describe the tomato fruit development by a sequence of models solved on a daily basis. This assumption was supported by a metabolic composition of the fruit varying very slowly compared with the duration of development and low changes of internal metabolites during 24 h (see for instance fumarate in Fig. S1). At steady‐state, the mass balance equation is expressed by

(Eqn 1) $$\frac{dX_{\mathrm{int}}}{dt}=NV=0.$$

With $X_{\mathrm{int}}={\left(x_{\mathrm{int},i}\right)}_{i\;=\;1\ldots m_{\mathrm{int}}}$ the vector of *m*_int_ internal metabolites, *V *= (*v*_*i*_)_*j *= 1…*n*_ the flux vector composed by the rates of *n* reactions of the network, $N={\left(n_{ij}\right)}_{i\;=\;1\ldots m_{\mathrm{int}},j\;=\;1\ldots n}$ the stoichiometry matrix where *n*_*ij*_ is the stoichiometric coefficient of metabolite *x*_int,*i*_ in reaction *j*.

A lower and an upper bound at each stage *s* (with *s *=* *10 … 50), denoted, respectively, by $V_{\min}^{s}={\left(v_{\min,j}^{s}\right)}_{j\;=\;1\ldots n}$ and $V_{\max}^{s}={\left(v_{\max,j}^{s}\right)}_{j\;=\;1\ldots n}$ such that the flux *V* satisfies:

(Eqn 2) $$V_{\min}^{s}\leq V\leq V_{\max}^{s},$$

which means $v_{\min,j}^{s}\leq v_{j}\leq v_{\max,j}^{s}$ for *j *=* *1…*n*.

Second, as detailed in Colombié *et al*. (2015) constraints were applied based on the thermodynamic properties of reversibility or irreversibility. Thus, among the 60 internal reactions of the metabolic network, 36 reactions were considered as irreversible (lower bounds set to zero, i.e. $v_{\min,j}^{s}=0$).

Third, the experimentally determined activities of 30 enzymes of central metabolism (Biais *et al*., 2014), expressed as mmol  per fruit d^−1^, were used to limit the corresponding flux, $v_{\max,j}^{s}$, in the metabolic network, considering that they correspond to maximal enzyme capacity. A regression‐fitted curve was built in order to give a constraint value at each day for modelling (see Table S2). The same values, but negatives, were used as lower bounds for reversible enzymes. When the capacity of a given enzyme was not known (< 20 reactions), the bounds were set to infinity.

Finally, from experimental concentrations of accumulated metabolites and biomass components, 16 fluxes (*n*_ext_) denoted by $v_{\mathrm{ext},k}^{s}$ (with *k *=* *0 …*n*_ext_ − 1), were evaluated on a daily basis throughout development using a previously described fitting method (Colombié *et al*., 2015) (see Table S2 for fitting parameters). Assuming that *V* is ordered (so that its *n*_ext_ last components *v*_*n−k*_ correspond to the outfluxes), to enforce these *v*_*k*_ to attain these fitted values *v*_ext,*k*_, we set:

$$v_{\min,n-k}^{s}=v_{\max,n-k}^{s}=v_{\mathrm{ext},k}^{s}\;\text{for}\;k=0\ldots n_{\mathrm{ext}}-1.$$

In order to take into account the variability of the experimental data, a bootstrap method was used to construct a number of resamples of the observed dataset obtained by random sampling with replacement from the original dataset. Once the transformation and the fit were applied to all of the resamples, a large set of polynomials was obtained. A 95% interval of prediction for the derivatives was used to fix the boundaries of the corresponding external fluxes denoted by $v_{\mathrm{ext}\_\mathrm{mini},k}^{s}\;\text{and}\;v_{\mathrm{ext}\_\mathrm{maxi},k}^{s}$. Thus, to calculate the variability of internal fluxes of the model, all 16 outfluxes were first fixed at the minimum $v_{\mathrm{ext}\_\mathrm{mini},k}^{s}$ for *k *=* *0…*n*_ext_ – 1, and then at the maximum $v_{\mathrm{ext}\_\mathrm{maxi},k}^{s}$ for *k *=* *0…*n*_ext_ − 1 (see Fig. S2).

In order to solve the system describing the development of the tomato fruit, the same objective function – that is, the minimization of the sum of the squares of all internal fluxes – was used for each stage in agreement with the principle of ‘minimal effort’ (minimal consumption of the available resources; Holzhutter, 2004).

The formulation of the problem is expressed as follows:

(Eqn 3) $$\text{minimize}\;f\left(V\right)=\sum_{j=1}^{n}{\left(v_{j}\right)}^{2},$$

at each *s* =* *10…50 subject to the constraints

NV=0,

$$V_{\min}^{s}\leq V\leq V_{\max}^{s}.$$

From a computational point of view, as *f* is a strictly convex function and the feasible set is convex this minimization leads to a unique solution at each stage (see the demonstration in Notes S2).

### Respiration and fluxes of energy dissipation peak at breaker stage

**Figure 1:**
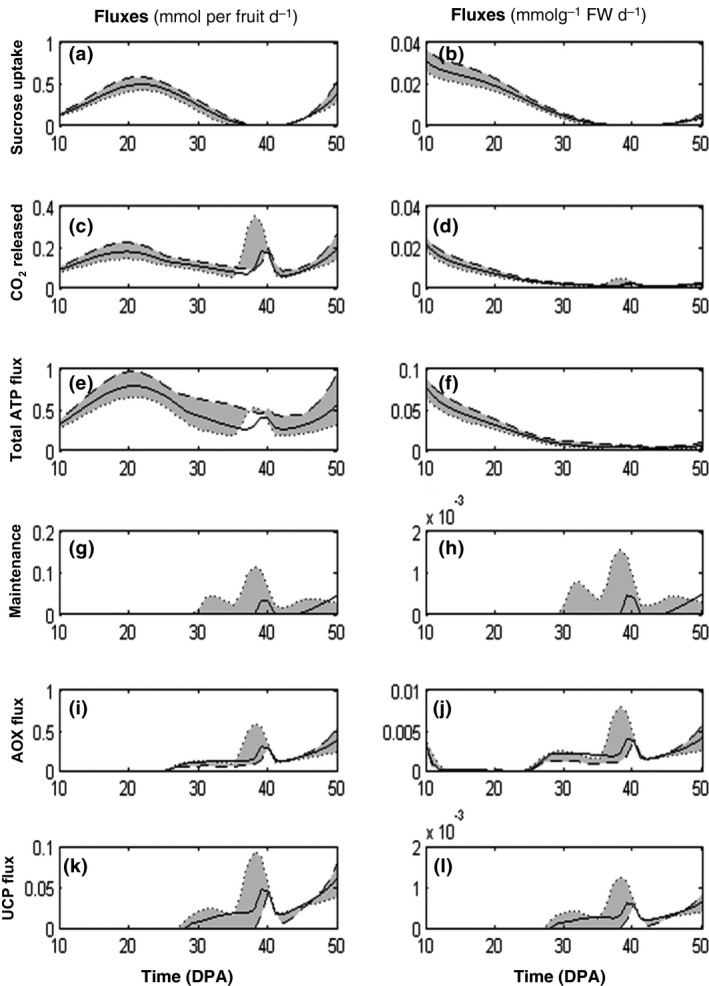
Time‐course of (a, b) sucrose uptake, (c, d) CO_2_ released and fluxes involved in tomato fruit (*Solanum lycopersicum)* energetic metabolism: (e, f) the total ATP flux, (g, h) ATP dissipated in maintenance, dissipating fluxes through (i, j) alternative oxidases (AOXs) and (k, l) uncoupling mitochondrial proteins (UCPs). Fluxes are expressed on a fruit basis (left panel) and on a g FW basis (right panel) with the model solved on a daily basis with best outfluxes (solid line), minima (dotted line) and maxima (dashed line) outfluxes considering a 95% interval of prediction from the biological variability. DPA, d post anthesis.

The system (Eqn (Eqn 3)) was solved each day of tomato fruit development and the calculated fluxes displayed profiles that were similar to those found previously (Colombié *et al*., 2015). Briefly, an increasing sugar metabolic activity throughout tomato fruit development and a drastic import of sucrose at the end of maturation supplying the vacuole sugar pools were observed when fluxes were expressed on the fruit‐basis. Conversely, when expressed on a gram basis, fluxes were very high in the very young fruit and decreased rapidly during cell expansion. Surprisingly, the improvement of the resolution, with 43 stages instead of nine as in Colombié *et al*. (2015) revealed a sharp peak at *c*. 40 DPA for a number of fluxes, especially fluxes involved in energy metabolism (ATP synthesis and consumption) and CO_2_ release from decarboxylation (CO_2_ was not balanced in the model (Fig. 1); note that the CO_2_ release was underestimated slightly due to the omission of CO_2_ produced by reactions for amino acids synthesis). This peak, which corresponds to high catabolic activity (*Vrcc1*, above all *Vrcc2*, also fluxes in TCA) with a high respiratory flux (CO_2_ released) was clearly visible when fluxes were expressed on a fruit basis, but also when fluxes were expressed on a gram basis (Fig. 1). This increase coincides with the respiration climacteric, which is known to occur just before ripening (Andrews, 1995). The calculated flux through AOXs and UCPs (*Vaox*,* Vucp*), which describes a part of energy dissipation, showed a similar profile with a progressive increase starting before 30 DPA and a peak at 40 DPA. The maintenance flux (*Vnga_ATP*) also displayed a peak at 40 DPA. Considering the experimental variability, an interval of flux prediction followed the same behaviour except for the total ATP flux and the maintenance for which the peak at 40 DPA tends to disappear when all outfluxes were maxima. To check whether the peak of energy dissipation found at 40 DPA was not due to the bypassing reactions added in the model through the alternative pathways, the two reactions *Vaox* and *Vucp* were removed. In consequence, flux profiles still displayed a high metabolic and respiratory activity at 40 DPA whereas the maintenance flux was clearly increased (Fig. S3). To sum up, the constraint‐based metabolic model predicted that a respiratory peak and a simultaneous dissipation of excess energy would occur at the breaker stage, and thus at the very moment of the occurrence of the respiration climacteric.

### At breaker stage, carbohydrate degradation results in excessive carbon supply

**Figure 2:**
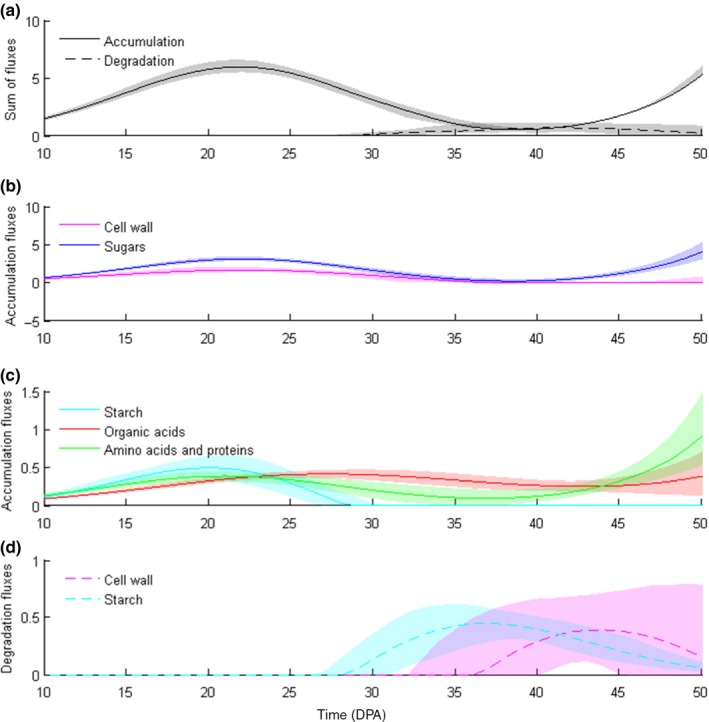
Time‐course of (a) total accumulation fluxes (solid line) and degradation fluxes (dashed line), (b, c) specific accumulation fluxes and (d) specific degradation fluxes. The coloured zone around each flux was calculated considering a 95% interval of prediction from the biological variability of tomato fruit (*Solanum lycopersicum)*. Fluxes are expressed in mmol per fruit d^−1^. DPA, d post anthesis.

**Figure 3:**
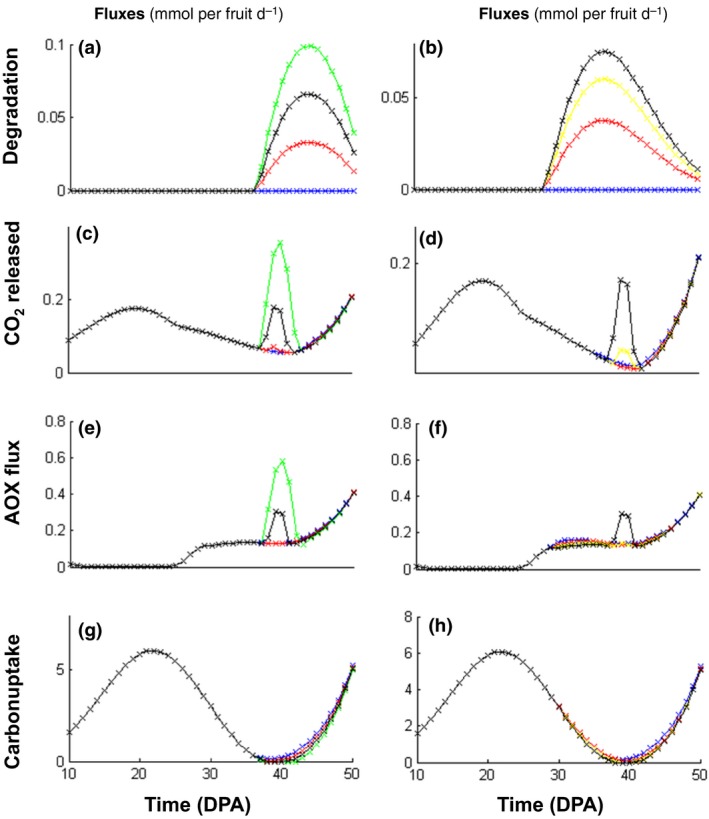
Impact of modulating (a, c, e, g) the cell wall degradation flux, (*Vcwd*: black, 100%; green, 150%; red, 50%; blue, 0%) and (b, d, f, h) the starch degradation flux (*Vsd*: black, 100%; yellow, 80%; red, 50%; blue, 0%) on (c, d) the calculated fluxes of CO_2_ released, (e, f) alternative oxidase and (g, h) carbon uptake with a daily basis resolution of the tomato fruit (*Solanum lycopersicum*) model and fluxes expressed in mmol per fruit d^−1^. DPA, d post anthesis.

At 40 DPA, fruit growth slowed down and the concentrations of major biomass components such as soluble sugars, organic acids and proteins reached a plateau. This resulted in a strong decrease in carbohydrates – sugars and cell wall – accumulation fluxes (Fig. 2). At the same time the sum of degradation fluxes of both starch and cell wall was at its maximum (Fig. 2). Indeed, between 30 and 50 DPA starch was totally degraded, together with a non‐negligible fraction of the cell wall, which implies that a large carbon pool was metabolized. This pool of carbon originating from both starch and cell wall reached 10 Cmmol per fruit at 50 DPA representing *c*. 7% of all the carbon stored at the end of fruit expansion (150 Cmmol per fruit at 50 DPA). To confirm that the origin of the energy peak observed at 40 DPA corresponded to the excess of degraded carbon polymers, we performed virtual experiments by increasing fluxes of both cell wall and starch degradation (Fig. 3). As expected, at 40 DPA the peak of CO_2_ released and energy dissipation of both maintenance and AOX increased when cell wall or starch degradation was increased artificially and, conversely, the peak decreased when these fluxes were decreased. Virtual experiments in which the fluxes towards sugars and organic acids were increased artificially *c*. 40 DPA were also performed. They resulted in a decrease up to a disappearance of the energy peak (data not shown). Taken together these results indicate that the degradation of stored carbon contributes substantially to the respiration climacteric. This explains why the variability calculated with all outfluxes at minimum increased the peak, whereas all outfluxes at maximum tended to make the peak ATP flux and maintenance disappear (Fig. 1).

### Stress conditions during tomato growth modulate the intensity of the metabolic peak

**Figure 4:**
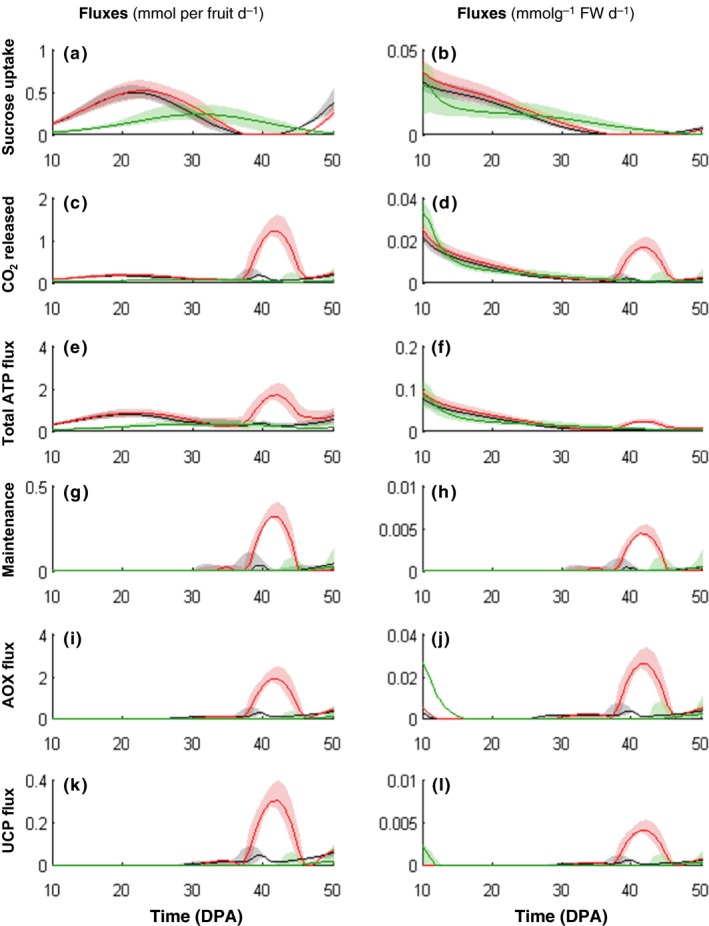
Time‐course of (a, b) sucrose uptake, (c, d) CO_2_ released, (e, f) total ATP flux, (g, h) ATP dissipated in maintenance, (i, j) alternative oxidase (AOX) flux and (k, l) uncoupling mitochondrial protein (UCP) flux. Fluxes are expressed on a fruit basis (left panel) and on a g FW basis (right panel) with the tomato fruit (*Solanum lycopersicum*) model solved on a daily basis using data obtained under control (black), water‐stressed (red) and shaded (green) conditions with best outfluxes (solid line), minima and maxima outfluxes considering a 95% interval of prediction from the biological variability. DPA, d post anthesis.

As previously reported the composition of the tomato fruit pericarp is not constant during fruit development and can be influenced by the environment (Davies & Hobson, 1981; Dorais *et al*., 2001). In particular, it is known that starch accumulation is increased upon water stressed and decreased under shaded conditions, in contrast to soluble sugars and organic acids, which were found to be affected only slightly by growth conditions (Biais *et al*., 2014). Because the amount of starch stored during fruit growth appeared to exert a major effect on the predicted energy burst, datasets obtained from fruits grown under these contrasting environmental conditions were implemented as constraints to run the model. Fruit growth under stress conditions was characterized (Fig. S4) and details of fruit composition were determined at nine stages of development (Fig. S5; Table S2). The main difference in biomass composition was found for starch and cell wall content. This was confirmed by a higher dry matter and carbon content under water‐stressed conditions and, conversely, a lower dry matter and carbon content under shaded conditions at 28 DPA (Table 1). As for control conditions, the accumulated metabolites and biomass components were used in order to set up the constraints of the flux balance model for both water‐stressed and shaded conditions, taking into account the experimental variability. After the daily basis resolution of the model, the selected calculated fluxes still displayed the peak of energy dissipation and CO_2_ production (respiration) (Fig. 4). However, the height of the energy burst was related to the amount of carbon degraded. Indeed under water‐stressed condition, higher starch content led to a higher peak than under control condition (Video S1); conversely under shaded conditions lower starch content led to peak attenuation. Interestingly, the origin of CO_2_ in the overall release, which was calculated as the sum of five fluxes (*Vidh*,* Vkgdh*,* Vpdh*,* Vme* and *Vg6pdh*) changed during development (Fig. S6). At the breaker stage, the CO_2_ from the TCA cycle (*Vidh +Vkgdh*) reached 30% and 50% of total CO_2_ released under control and water‐stressed conditions, respectively, at the expense of *Vpdh* and *Vg6pdh*. Finally, cell wall degradation took place under all conditions and both galactose and galacturonate, two intermediates of cell wall degradation, were accumulated at the last stages of development, after 49 DPA (Fig. S7). As expected and according to the data fits, cell wall degradation started before galactose and galacturonate would accumulate (Fig. 2). Then a rapid estimation based on the amount of degraded cell wall suggests that much higher amounts of these intermediates are released than measured (i.e. 1.2 vs 0.1 mg g^−1^ FW detected by NMR). Taken together these results: (1) confirm that cell wall and starch degradation supplies carbon for the respiration climacteric, and (2) show that environmental growth conditions, that is, water stress and shading, modulate the intensity of the energy peak observed before tomato fruit ripening.

**Table 1:** Tomato fruit and pericarp dry matter, carbon (C) and nitrogen (N) contents (% DW) of fruit pericarp measured under control, water‐stressed and shaded conditions at 28 d post anthesis (DPA). Values are expressed as mean ± SD

	Control	Water stressed	Shaded
Fruit DW %	6.11 ± 1.67	6.98 ± 2.00	4.96 ± 0.54
Pericarp DW %	6.81 ± 1.12	8.64 ± 1.37	5.83 ± 1.16
% C	41.41 ± 0.19	42.06 ± 0.20	40.77 ± 0.05
% N	1.41 ± 0.18	1.45 ± 0.08	1.62 ± 0.15

**Figure 5:**
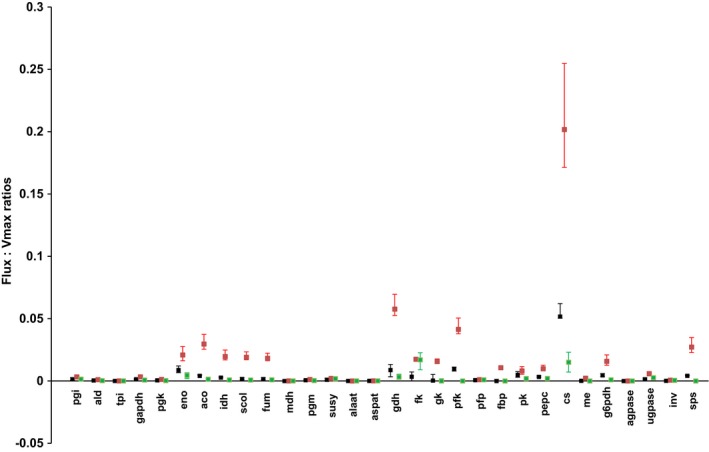
Flux : capacity ratios at 40 d post anthesis (DPA) under control (black), water‐stressed (red) and shaded (green) conditions. Ratios calculated for 30 enzymes for which the capacity was determined experimentally with fluxes calculated with best outfluxes (square), minima and maxima outfluxes considering a 95% interval of prediction from the biological variability.

Finally, for 30 fluxes, ratios between calculated fluxes and enzymatic capacities were calculated to estimate how much enzyme capacity is readily involved in a given metabolic flux. As previously reported for tomato fruit, most of these ratios have been shown to be very small (Beauvoit *et al*., 2014; Colombié *et al*., 2015), indicating that the pool of enzymes largely exceeds the catalytic demand for biosynthetic and accumulation processes. Highest ratios were found under water‐stressed condition at 40 DPA (Fig. 5). Thus, the citrate synthase flux was the highest, reaching 20–25% of the enzyme capacity, whereas phosphofructokinase and glutamate dehydrogenase fluxes reached 5% of their respective capacities.

### Does the excess of energy dissipation lead to fruit thermogenesis?

We evaluated whether the metabolic reactions involved in the respiration climacteric could lead to a significant increase in fruit temperature. As the fruit temperature is linked to its heat budget and varies with the gain or loss of energy, we estimated independently both sources of energy change, metabolic activities and physical events linked with environment.

Assuming that in aerobic and nonfermentative tissue (i.e. O_2_ : CO_2_ ratio equals one) the main exothermic reaction is respiration; we estimated the heat production rate (*Q* in W or J s^‐1^) per fruit as follows (Fig. S8):

(Eqn 4) $$Q=-V_{{\mathrm{CO}}_{2}}.\Delta rH^{\prime 0},$$

($V_{{\mathrm{CO}}_{2}}$, flux of CO_2_ release (in mmol per fruit s^−1^); and ∆*r H*′^0^, molar reaction enthalpy of NADH‐linked O_2_ reduction (i.e. −450 kJ mol^−1^; Dejean *et al*., 2001)).

**Figure 6:**
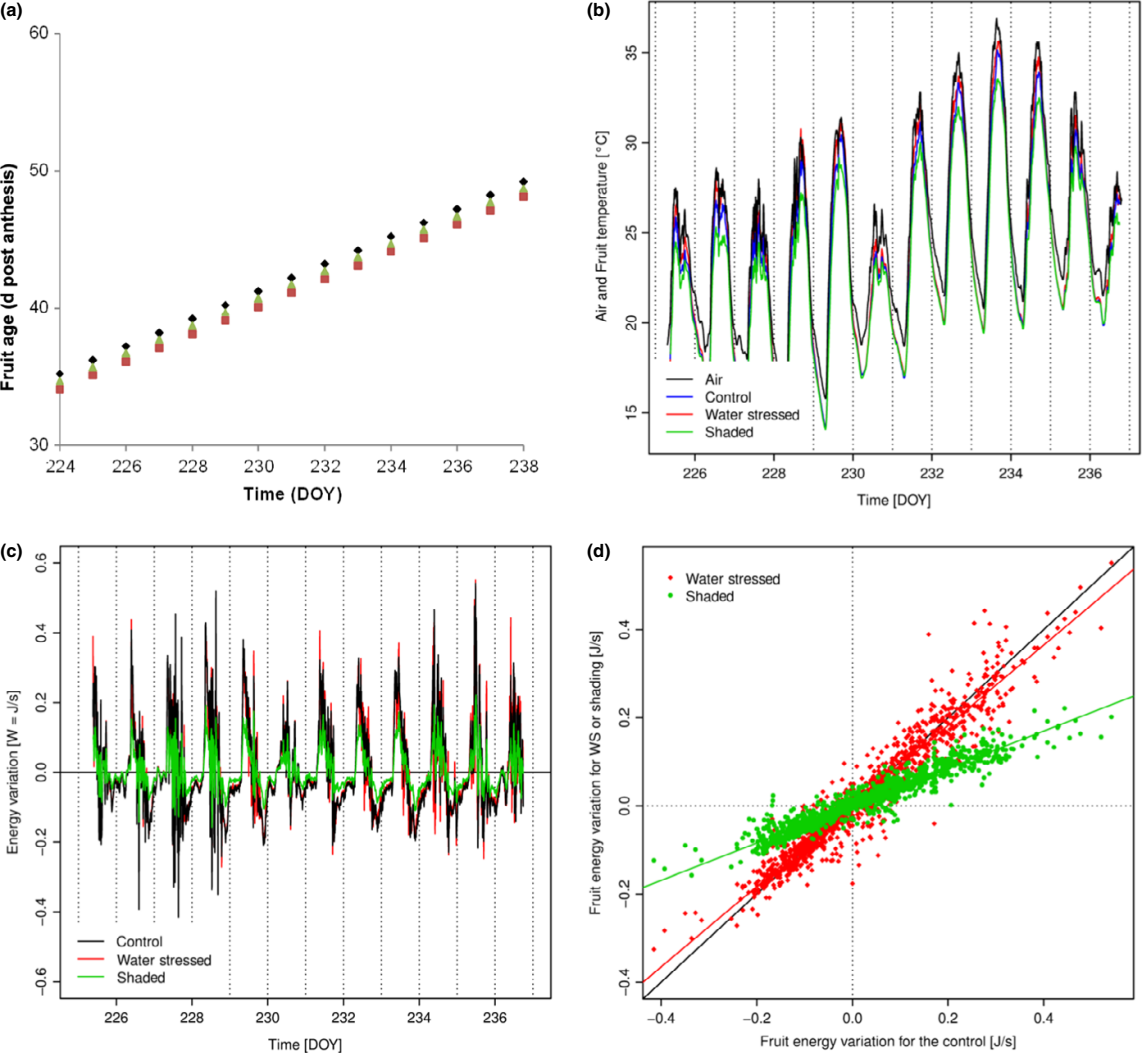
(a) Tomato fruit (*Solanum lycopersicum)* age expressed in d post anthesis (DPA), (b) air and median fruit temperatures and (c) median fruit energy variation vs time (days of year, DOY) under control (black), water‐stressed (red) and shaded (green) conditions and (d) a scatter plot of fruit energy variation for the first temperature recording period.

The fruit heat variation due to the environment was calculated, knowing the fruit volume, its density and estimating the specific heat capacity, as proposed by Sahin & Sumnu (2006). Fruit and air temperature displayed similar profiles but fruit temperatures were higher than air temperature under control and even more under water‐stressed conditions. Under shaded conditions, fruit temperature was always lower than air temperature (Fig. 6b). Large fruit heat variations (gain or loss) occured early in the morning and late in the afternoon, that is, when large air temperature variations are observed. As the shaded net buffered diurnal air temperature variations with time, fruit heat variations were lower in this treatment than under control and water‐stressed conditions (Fig. 6c). As variation of temperature was quite similar for the three recording periods, fruit heat variations were similar whatever the age of the fruits (Fig. S9).

Finally, with a maximal estimated heat produced by metabolic activities close to 0.7 × 10^−3^ W at 40 DPA under water‐stressed condition (Fig. S8), it was about thousand times lower than the fruit heat variation due to environment (*c*. 0.6 W). Thus, the tomato fruit thermogenesis due to the respiration climacteric appeared negligible.

## Discussion

In this work, an increase of the resolution of the time step and an improvement of the energy dissipation description in the model described in Colombié *et al*. (2015) enabled us to reveal a respiratory peak and an energy burst, which appeared as an emerging property of the metabolic model. In particular, the peak was precisely positioned at 40 d post anthesis (DPA) – that is, just before ripening – thus coinciding with the respiratory climacteric (Chalmers & Rowan, 1971). This result pointed out that an appropriate biochemical description of the metabolic network gives an accurate predictive power of constraint‐based modelling for respiration (Sweetlove *et al*., 2013). This offered us the opportunity to improve our understanding of the relatively poorly known physiology of a phenomenon that is observed in a range of fleshy fruit species.

### Starch and cell wall degradation fuels the respiration climacteric of tomato fruit

In the model, two main energy dissipation processes were implemented by bypassing: (1) the cytochrome pathway of miETC (i.e. alternative oxidases (AOXs)) and/or (2) the proton‐linked oxidative phosphorylation (i.e. plant uncoupling mitochondrial proteins (UCPs)). Moreover, the nongrowing associated ATP maintenance (*Vnga‐ATPm*) was still present to balance energy cofactors and consequently can be considered as an adjustment variable. Energy costs of maintenance (e.g. protein turnover and conservation of the electrochemical potential difference across plasmalemma and tonoplast) can vary widely depending on the tissue, the growth conditions or the developmental stage. The increase in ATP, which in many fruits occurs during the respiration climacteric (Brady, 1987), results from the supply of energy in excess of the demand. Energy balance and energy use in nongrowing tissues (called the maintenance energy requirement) are relatively poorly understood. In the present study, the energy peak was found to result from an excessive supply of carbon from the rapid degradation of both starch and cell wall polysaccharides, whereas growth and hence carbon demand dropped. This result was then validated by water‐stressed and shading experiments, in which carbon storage during fruit growth was (respectively) increased and decreased. Although the link between starch degradation and respiration climacteric in fruits has already been proposed in, for example, banana (Seymour, 1993), kiwifruit (Han & Kawabata, 2002) and apple (Thammawong & Arakawa, 2007), it is interesting to note that cell wall degradation substantially contributes to the energy peak. Cell wall remodelling at ripening is well documented. In tomato, the most widely reported changes in wall structure are an increase in soluble polyuronide and a decrease in galactose and arabinose residues (Seymour *et al*., 1990). The present study showed that such degradation represents 40% of the carbon in the respiration climacteric.

### AOXs and UCPs partially buffer excessive energy released during the respiration climacteric

At the breaker stage the peak of the AOX flux is in agreement with previous results on tomato (Xu *et al*., 2012). For UCPs, studies on fruit ripening have shown that its expression is tuned to physiological conditions (Vercesi *et al*., 2006). However, in tomato the amount and activity of UCPs were shown to decrease during the later stages of postharvest fruit ripening (Almeida *et al*., 1999), whereas the flux calculated here was still increasing at the end of development.

It is possible that UCPs and AOX pathways operate with different efficiencies under distinct physiological conditions. Interestingly, with increasing fatty acids concentration in post‐growth stages – like during fruit ripening but perhaps also during senescence and flowering, UCP activity could reach maximum velocity whereas AOX activity would be switched off (Jarmuszkiewicz *et al*., 2001). Our results for fatty acids showed increases in linoleic acid and especially linolenic acid after 40 DPA. Together with other fatty acids (myristic acid, palmitic acid), they have been shown to activate AtPUMP1, the recombinant Arabidopsis UCP (Vercesi *et al*., 2006). Conversely, as suggested by *in vitro* studies (Rhoads *et al*., 1998), accumulation of pyruvate, which is likely to occur in tomato during ripening (Chalmers & Rowan, 1971) could be a regulatory factor increasing the activity of AOX at the expense of the cytochrome pathway in early stages of ripening, whereas UCPs would be functionally silent (Jarmuszkiewicz *et al*., 2001).

There is a consensus in the literature about the involvement of AOXs and UCPs in stress resistance in plants (Blokhina & Fagerstedt, 2010). Their activity is thought to limit the production of reactive oxygen species (ROS), which results from the imbalance between energy supply and demand under stress. Results obtained in the present study confirm this role, because fruits grown under water stressed accumulate more starch during their growth, thus leading to a massive energy release at breaker stage. More than 30 yr ago, Fukushima *et al*. (1980) studied the relationship between water status and climacteric crisis in several fruit species including tomato. These authors found a correlation between post‐harvest respiration and turgor pressure, which has never been investigated further. This contrasts with mango fruits, in which the expression of AOXs and UCPs was investigated during ripening by Considine *et al*. (2001). These authors showed that the abundance of AOX transcripts and proteins both peaked at the ripe stage whereas expression of the UCP single gene peaked at the turning stage and the protein abundance peaked at the ripe stage. Because both transcripts and proteins for the AOXs and UCPs increased in a similar pattern, they suggested that their gene expression was not controlled and may be active simultaneously with increases in cytochrome chain components facilitating the climacteric burst of respiration. Tsaniklidis *et al*. (2014) recently described considerable differences in the regulation of AOX between seeded and parthenocarpic fruits during tomato fruit development. These authors suggested that all AOX isoenzymes studied so far are involved in fruit metabolism, particularly during the climacteric rise of respiration. AOX gene transcript accumulation was higher in seeded fruits in most stages of fruit development. This fact combined with the higher respiration rates of seeded fruit indicates their higher metabolic needs during the maturation process and the need for AOX activity during seed development. Taken together, these facts suggest that AOXs and UCPs may play more than one role during fruit development.

### The respiration climacteric is enabling rapid reprogramming of metabolism at ripening

The model‐predicted increase in respiratory fluxes is in agreement with a previous study, in which the role of AOXs was studied in fruits of transgenic tomato plants (Xu *et al*., 2012). Interestingly, fruits with reduced AOX capacity showed no respiration climacteric and failed to ripen after application of 1‐methylcyclopropene, which blocks ethylene signalling. In isolated mitochondria, AOX activity has been shown to be stimulated in a feed‐forward mode by pyruvate and NADPH (Vanlerberghe, 2013; Perotti *et al*., 2014), which are likely to increase at high glycolytic and oxidative pentose phosphate pathway fluxes, respectively, suggesting that the increased AOX flux is a consequence of the significant release of stored carbon in the cell.

In the present study, the model found no energy peak in fruits grown under shade (Fig. 4), which suggests that less energy is available for processes taking place at the onset of ripening. Furthermore, ripening was significantly delayed in these fruits although their growth reached a plateau at the same stage (40 DPA) as under control or water‐stressed conditions. Given that protein synthesis, which is a high energy consuming process, starts again at exactly this point in many climacteric fruit species (Brady, 1987), it can be hypothesized that the respiration climacteric acts as a booster of the reprogramming orchestrated by ethylene. Indeed, signalling through the plant hormone ethylene remains the most well‐defined pathway that mediates the phenotypic changes that occur during ripening. Increased respiration provides the ATP required for the methionine cycle and can lead to high rates of ethylene production without high levels of intracellular methionine (Barry & Giovannoni, 2007).

### Fruit thermogenesis is not significant at the breaker stage

The non‐energy conserving property of AOXs and UCPs results in a lower ATP yield and consequently, in a larger respiratory activity thus leading to heat production also called thermogenesis, which has been observed in several plant species. The most dramatic examples of plant thermogenesis are in the *Araceae* (Zhu *et al*., 2011) and the apparent increase in temperature observed in fruit species was very low in comparison to that observed in thermogenic spadices. For instance, in mango a rise of nearly 10°C in temperature during the respiration climacteric has been reported (Kumar *et al*., 1990). In banana fruits, which degrade massive amounts of starch during ripening (starch represents > 20% of the dry matter in unripe bananas), the involvement of alternative respiration resulted in an increase of 4°C, which was associated with an increase of *c*. 20% in total respiration and little change in AOX capacity (Kumar & Sinha, 1992). As far as we know, thermogenesis of the tomato fruit has not been reported and here we provide evidence that the heat released by metabolic activities was much lower than the daily heat variations generated by energy exchanges by radiation, transpiration, conduction and convection at the tomato fruit surface. This result is in agreement with Jarmuszkiewicz *et al*. (2001) who recognized the only obvious physiological function of AOXs and UCPs in specialized plant thermogenic tissues for heat generation. These authors suggested that in nonthermogenic tissue, heat production was a side event (not a useless event) of free‐energy dissipation where AOX and UCP activities may have a regulatory role in the balance of energy metabolism (Jarmuszkiewicz *et al*., 2001).

Thus, it was not surprising that in the present study, whereas fruits grown under water stress displayed higher AOX and UCP fluxes, they did not ripen faster.

### Conclusion

Climacteric behaviour has been widely studied in fruits but the corresponding fruit respiration is difficult to measure. Using a constraint‐based model describing the cytochromic respiration and its bypasses, we predicted metabolic fluxes of tomato fruit development on a daily basis. The model revealed a peak of CO_2_ release at the breaker stage and the simultaneous dissipation of excess energy, supporting the concept of respiration climacteric. We demonstrated that: (1) this peak is due to unbalanced carbon allocation just before ripening, (2) environmental stress modulates the quantity of carbon stored and hence the intensity of fruit respiration climacteric, and (3) heat from the metabolic activity of respiration contributes negligibly to fruit temperature change. Our constraint‐based metabolic modelling proved to be a good tool with which to estimate fruit respiration, which is difficult to measure in fruits still attached on the mother plant.

In order to go further in developing this tool, as there are a number of fleshy fruit species, including tomato, with different cultivars exhibiting climacteric or nonclimacteric behaviour (Barry & Giovannoni, 2007), it will be important to transfer and adapt the present model to nonclimacteric genotypes (mutants or transformants) and to various other climacteric and nonclimacteric species than tomato. In this way we hope to attain a better understanding of the physiological meaning of the respiration climacteric.

## Author contributions

S.C. designed and wrote the paper; B.Be. wrote the respiration and energy reactions in the model; C.N. calculated the constraints and solved the model; C.B. performed the culture; G.V. calculated the fruit energy changes; B.Bi. performed enzyme and metabolite analyses; S.L.G. analysed the cell wall; C.C. analysed the traits of the culture; M.M. and A.M. performed NMR analyses; S.B. performed amino acid analyses; M.D‐N. drew the flux maps; J‐P.M. participated in result analysis; and Y.G. supervised the work and co‐wrote the discussion.

## Supporting information

Please note: Wiley Blackwell are not responsible for the content or functionality of any Supporting Information supplied by the authors. Any queries (other than missing material) should be directed to the *New Phytologist* Central Office.

**Fig. S1 **Fumarate concentration throughout tomato fruit development.
**Fig. S2 **Fit of the accumulated metabolites and biomass compounds.
**Fig. S3 **Importance of dissipating fluxes, AOX and UCP, on calculated fluxes.
**Fig. S4 **Plant culture and fruit characterization.
**Fig. S5 **Fruit composition throughout development.
**Fig. S6** Origin of CO_2_ released throughout tomato fruit development.
**Fig. S7 **Galactose and galacturonate throughout the tomato fruit development.
**Fig. S8 **Estimated heat from metabolic activities.
**Fig. S9 **Fruit temperature and fruit energy variations.
**Notes S2** Mathematical demonstration of unicity.

**Table S1** Model description

**Table S2 **Raw data and fits

**Notes S1** Stoichiometric model in *sbml*.

**Video S1 **Flux maps of water stress compared with control.
