# Supplementary material for: Respiration climacteric in tomato fruits elucidated by constraint‐based modelling
Source: New Phytol. 2016 Nov 15;213(4):1726–39. doi: 10.1111/nph.14301 (PMC6079640; doi:10.1111/nph.14301)
Supplement: Supplementary file 1 — Fig. S1 Fumarate concentration throughout tomato fruit development. Fig. S2 Fit of the accumulated metabolites and biomass compounds. Fig. S3 Importance of dissipating fluxes, AOX and UCP, on calculated fluxes. Fig. S4 Plant culture and fruit characterization. Fig. S5 Fruit composition throughout development. Fig. S6 Origin of CO2 released throughout tomato fruit development. Fig. S7 Galactose and galacturonate throughout the tomato fruit development. Fig. S8 Estimated heat from metabolic activities. Fig. S9 Fruit temperature and fruit energy variations. Notes S2 Mathematical demonstration of unicity. [file NPH-213-1726-s001.pdf]

New Phytologist

New Phytologist Supporting Information Figs S1–S9 and Notes S2

Article title: Respiration climacteric in tomato fruits elucidated by constraint-based modelling

Authors: Sophie Colombié, Bertrand Beauvoit, Christine Nazaret, Camille Bénard, Gilles Vercambre, Sophie Le Gall, Benoit Biais, Cécile Cabasson, Mickaël Maucourt, Stéphane Bernillon, Annick Moing, Martine Dieuaide-Noubhani, Jean-Pierre Mazat and Yves Gibon

Article acceptance date: 22 September 2016

The following Supporting Information is available for this article:

Fig. S1 Fumarate concentration throughout tomato fruit development.

Fig. S2 Fit of the accumulated metabolites and biomass compounds.

Fig. S3 Importance of dissipating fluxes, AOX and UCP, on calculated fluxes.

Fig. S4 Plant culture and fruit characterisation.

Fig. S5 Fruit composition throughout development.

Fig. S6 Origin of CO_2 released throughout tomato fruit development.

Fig. S7 Galactose and galacturonate throughout the tomato fruit development.

Fig. S8 Estimated heat from metabolic activities.

Fig. S9 Fruit temperature and fruit energy variations.

Table S1 Model description (separate Excel file)

Table S2 Raw data and fits (separate Excel file)

Notes S1 Stoichiometric model in *sbml* (separate .xml file).

Notes S2 Mathematical demonstration of unicity.

Video S1 Flux maps of water stress compared to control (separate .mp4 file).

Fig. S1 Fumarate concentration throughout tomato fruit development. Time-course of fumarate content in tomato fruit pericarp measured by ^{1}H-NMR throughout the tomato fruit development and every 3 h (zoom in the box) from 24 to 25 days post anthesis.

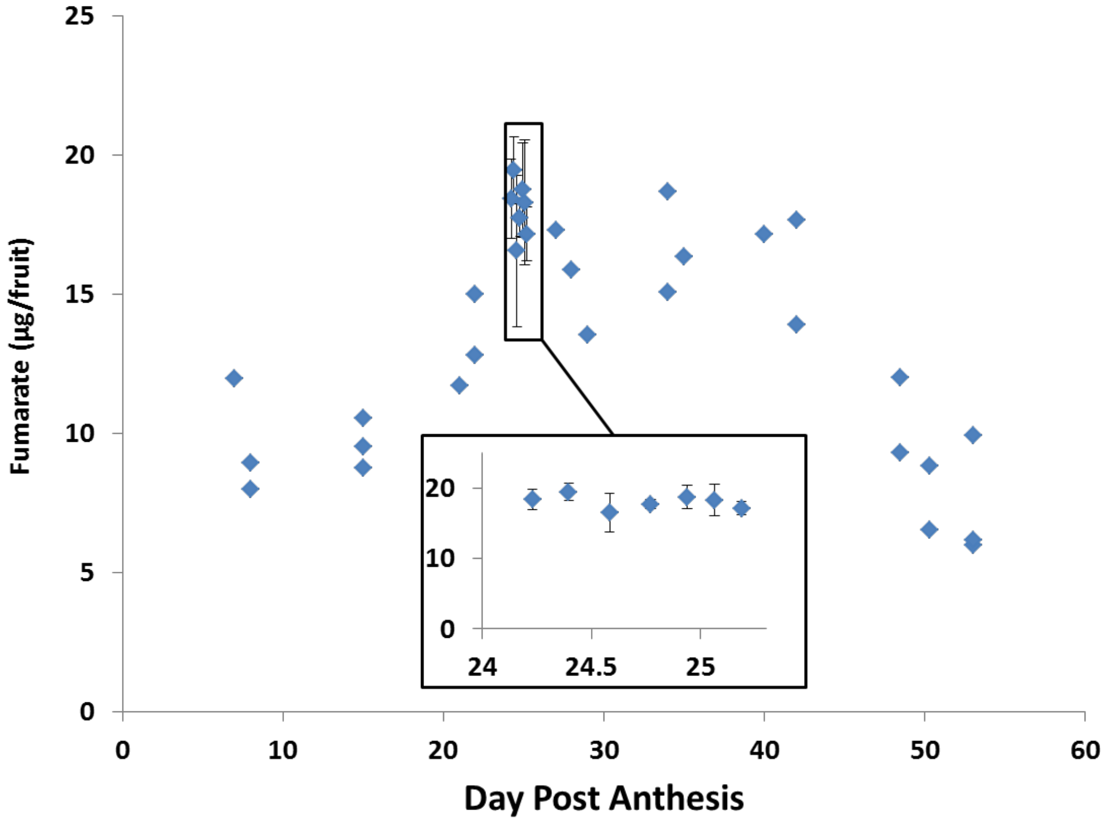

Fig. S2 Fit of the accumulated metabolites and biomass compounds**.** Time-course of the 16 accumulated metabolites and biomass compounds (left panel) and the corresponding flux calculated by derivative of the best fit (solid line), minima and maxima outfluxes considering a 95% interval of prediction from the biological variability (dotted lines) under control (blue), water stress (red) and shading (green) conditions .

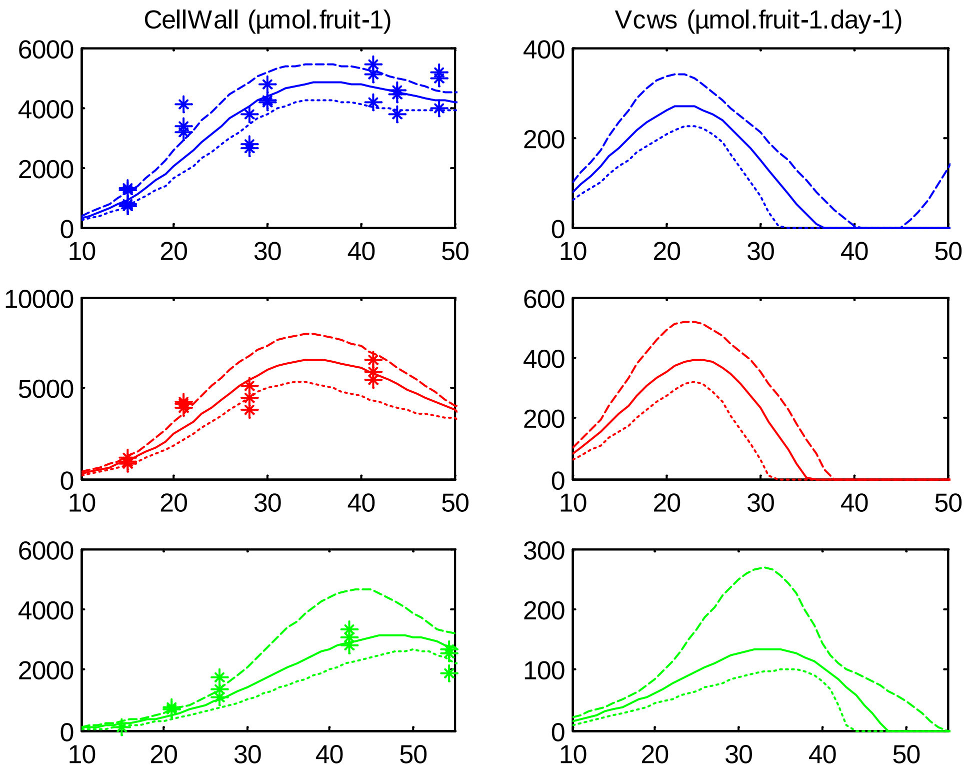

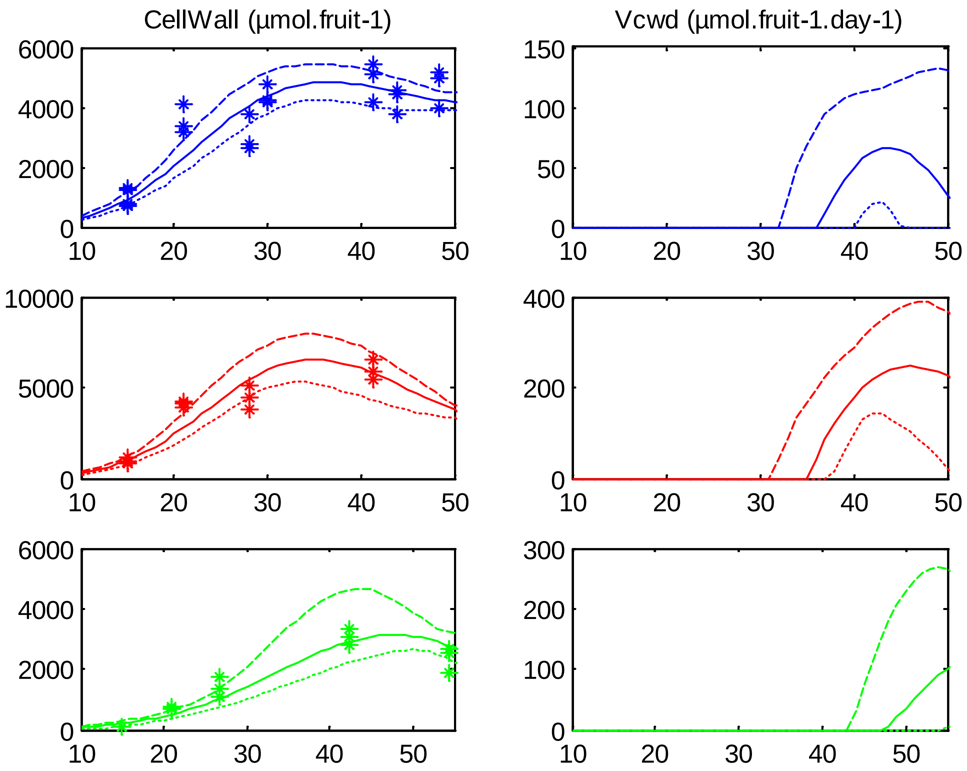
CellWall (µmol.fruit-1)
Vcwd (µmol.fruit-1.day-1)
0
2000
4000
6000
10000
5000
50
100
150
200
300
400
10
20
30
40
50

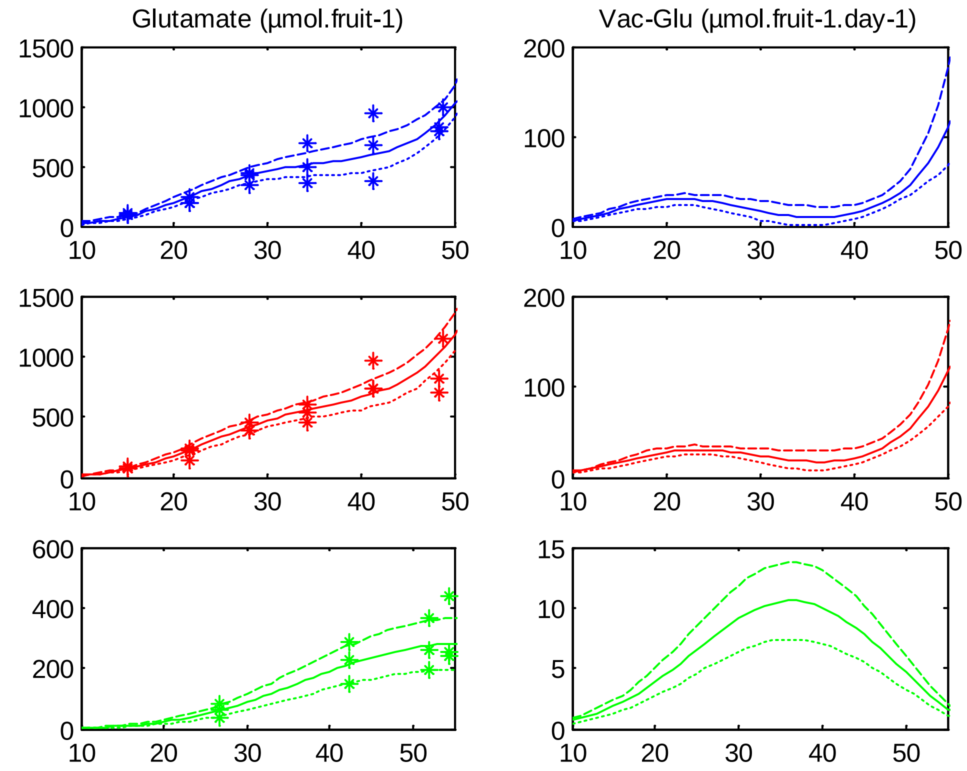
Glutamate (µmol.fruit-1)
Vac-Glu (µmol.fruit-1.day-1)
0
500
1000
1500
200
400
600
100
5
10
15
20
30
40
50

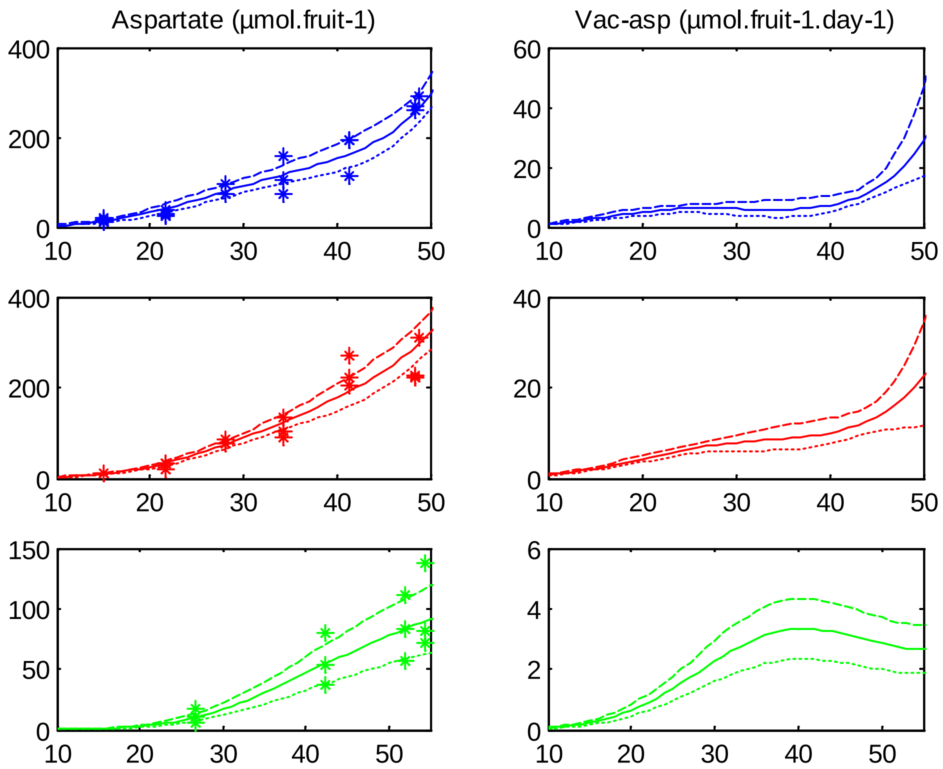

Aspartate (µmol.fruit-1)
Vac-asp (µmol.fruit-1.day-1)

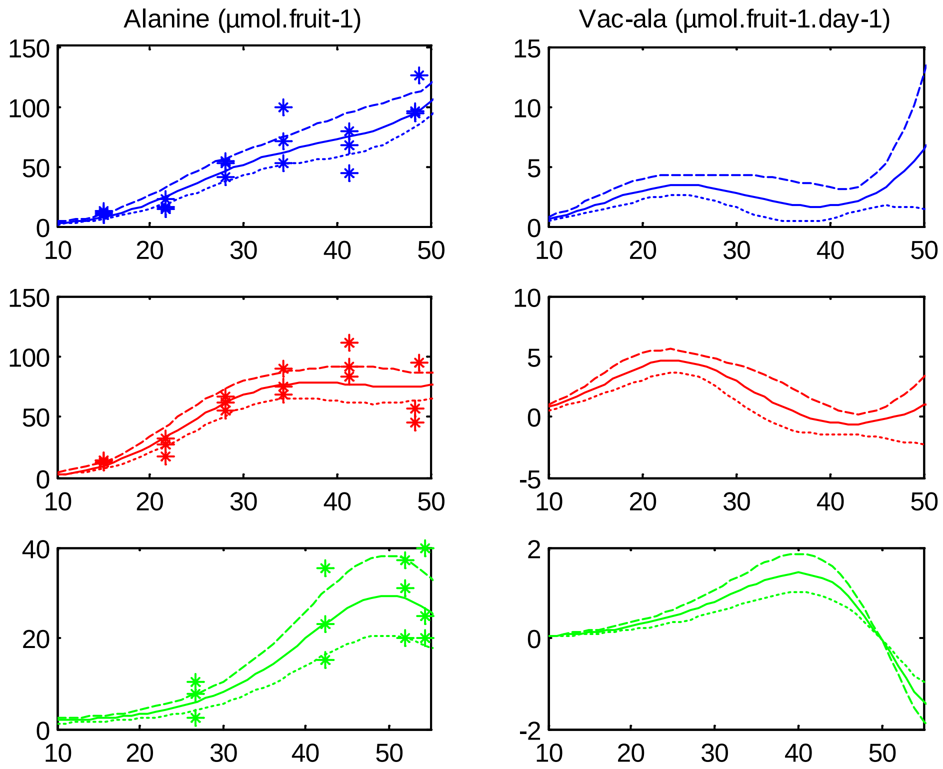

Alanine (µmol.fruit-1)
Vac-ala (µmol.fruit-1.day-1)

New Phytologist

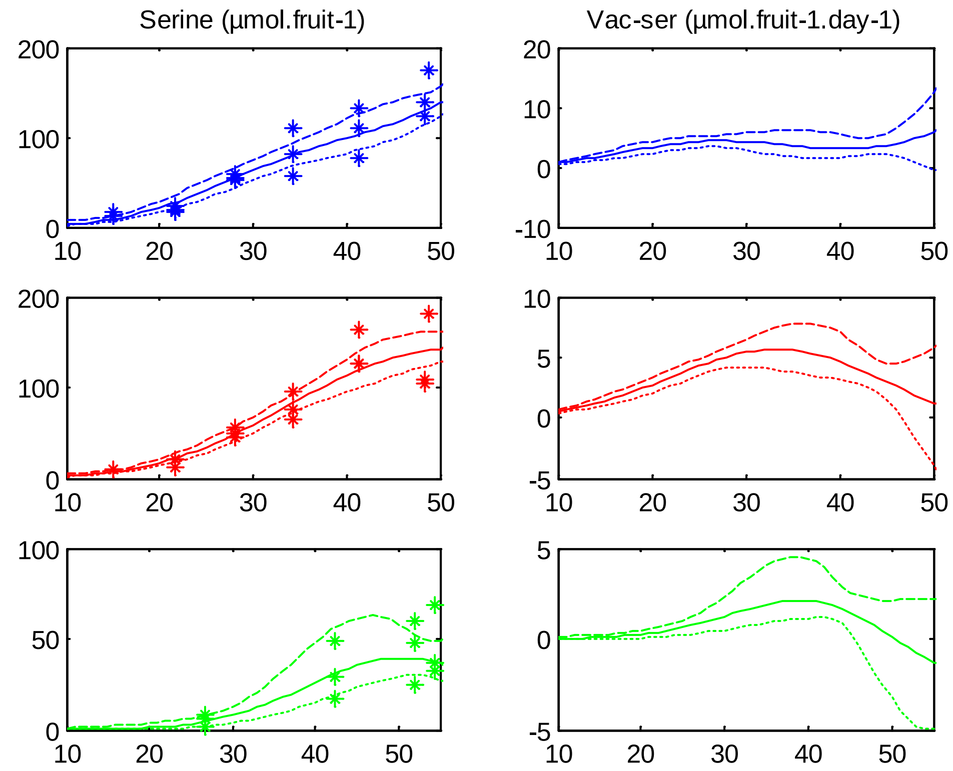

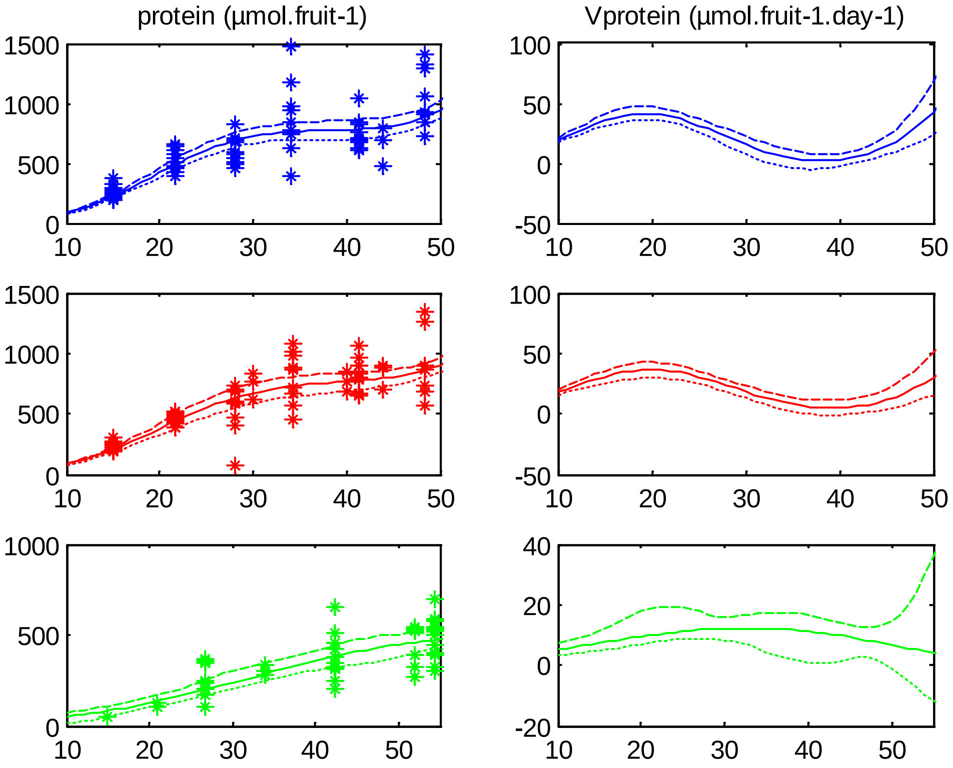

New Phytologist

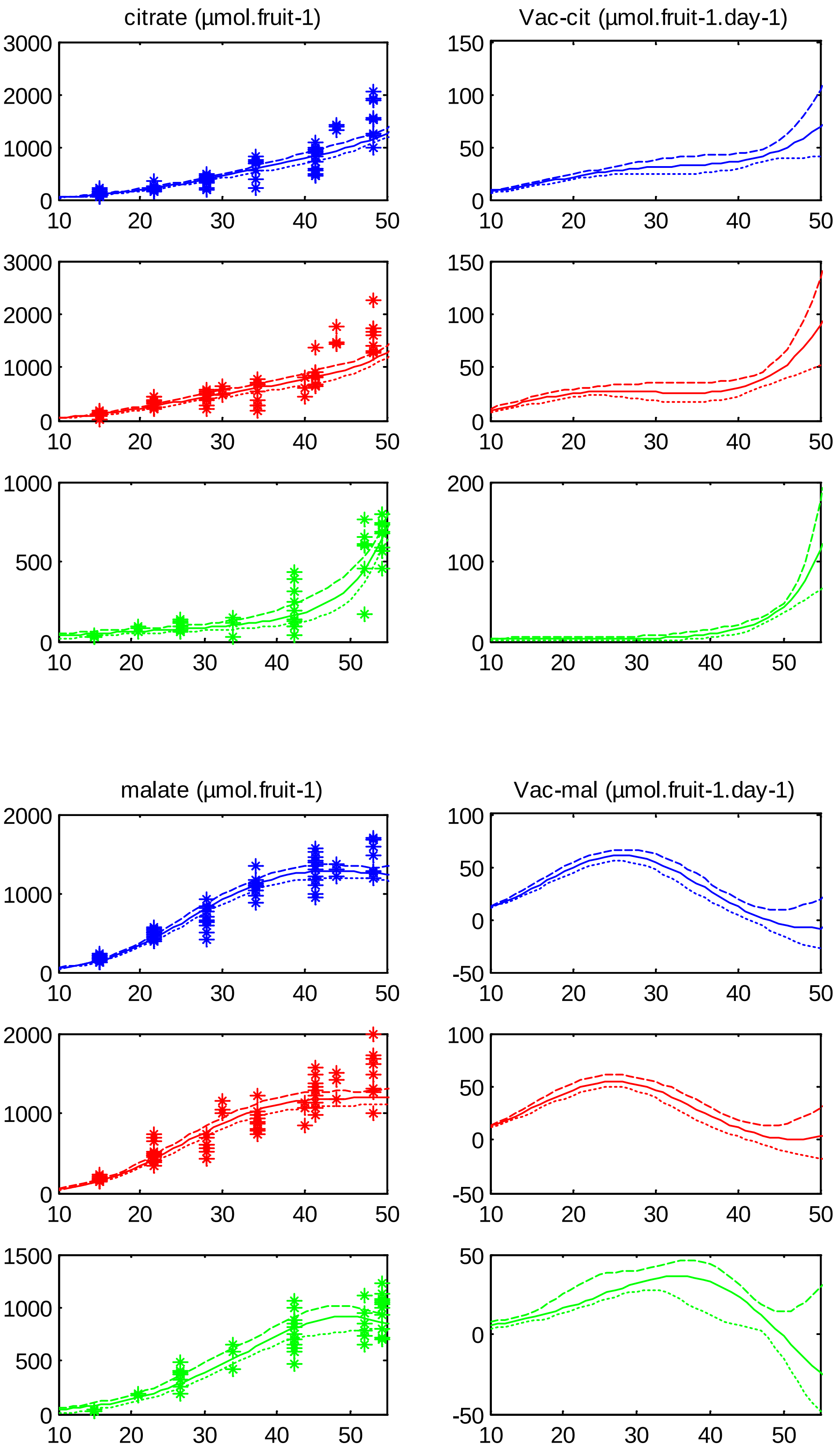

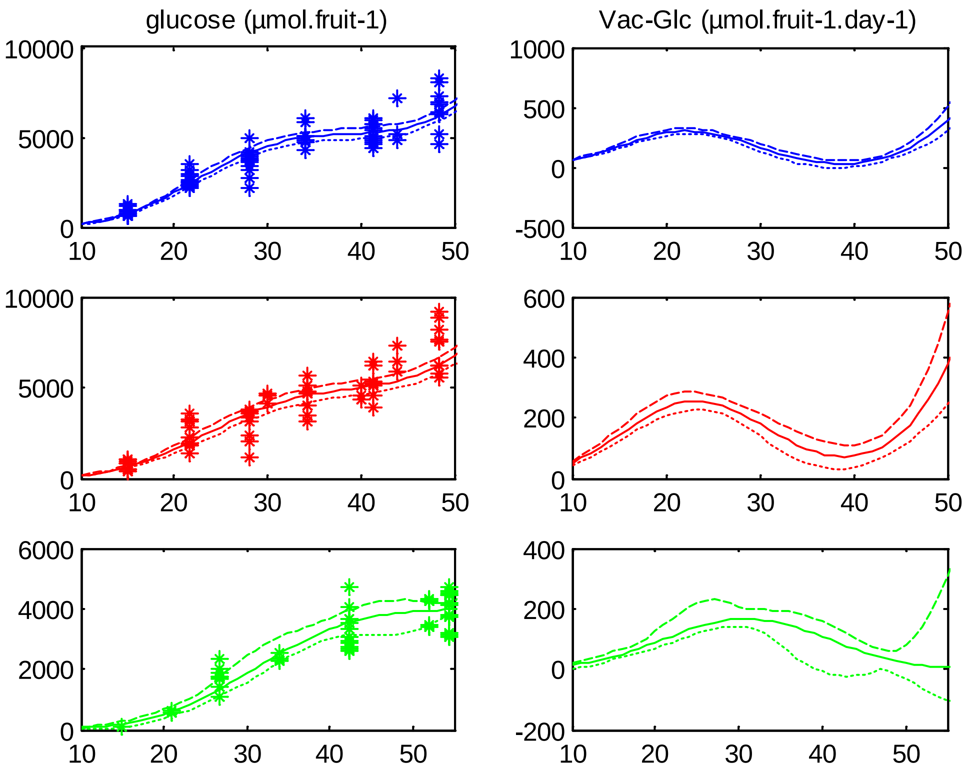
glucose (µmol.fruit-1)
Vac-Glc (µmol.fruit-1.day-1)
10000
5000
0
1000
500
0
-500
10
20
30
40
50
10000
5000
0
600
400
200
0
6000
4000
2000
0
400
200
0
-200

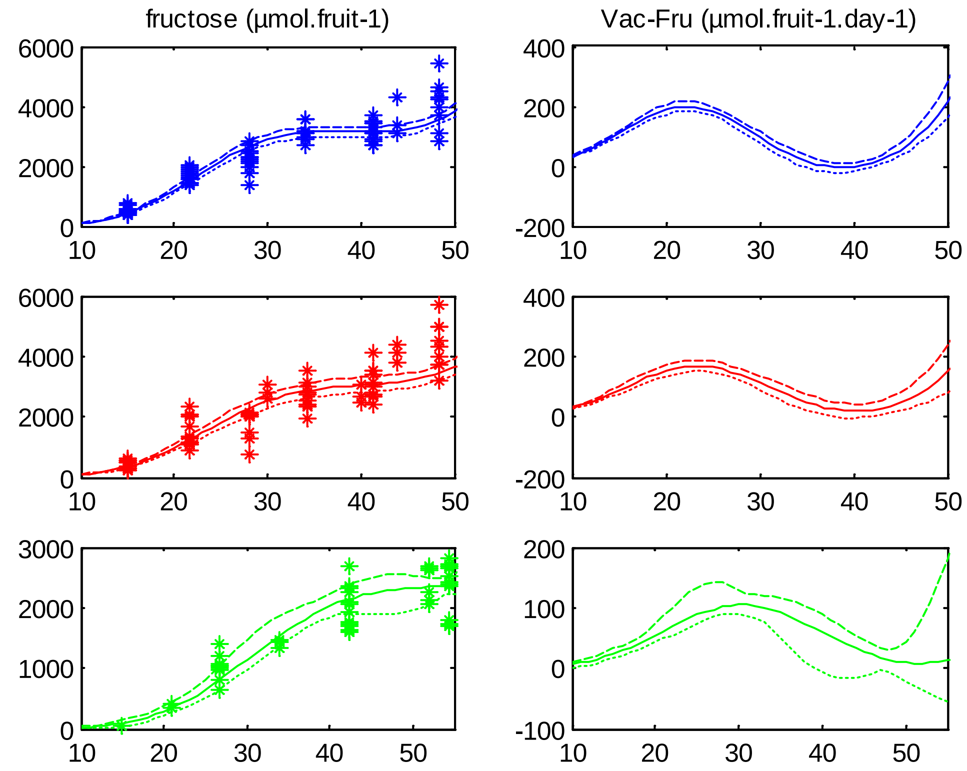
fructose (µmol.fruit-1)
Vac-Fru (µmol.fruit-1.day-1)
6000
4000
2000
0
400
200
0
-200
10
20
30
40
50
3000
2000
1000
0
200
100
0
-100

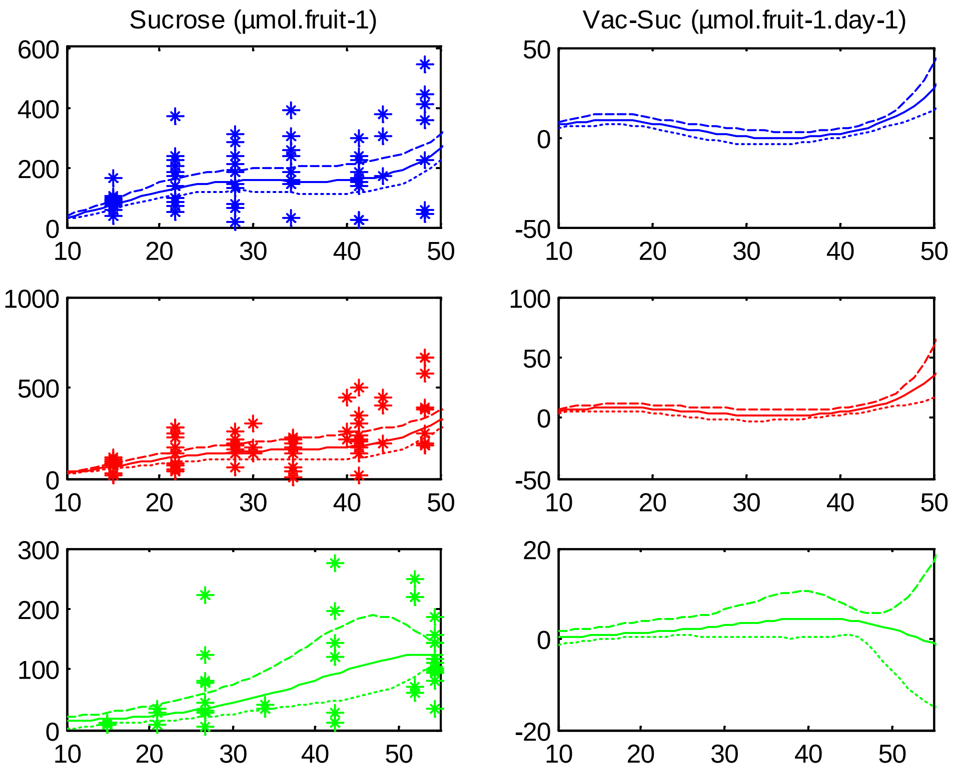
Sucrose (µmol.fruit-1)
Vac-Suc (µmol.fruit-1.day-1)
600
400
200
0
50
0
-50
1000
500
100
300
100
20
-20
10
20
30
40
50

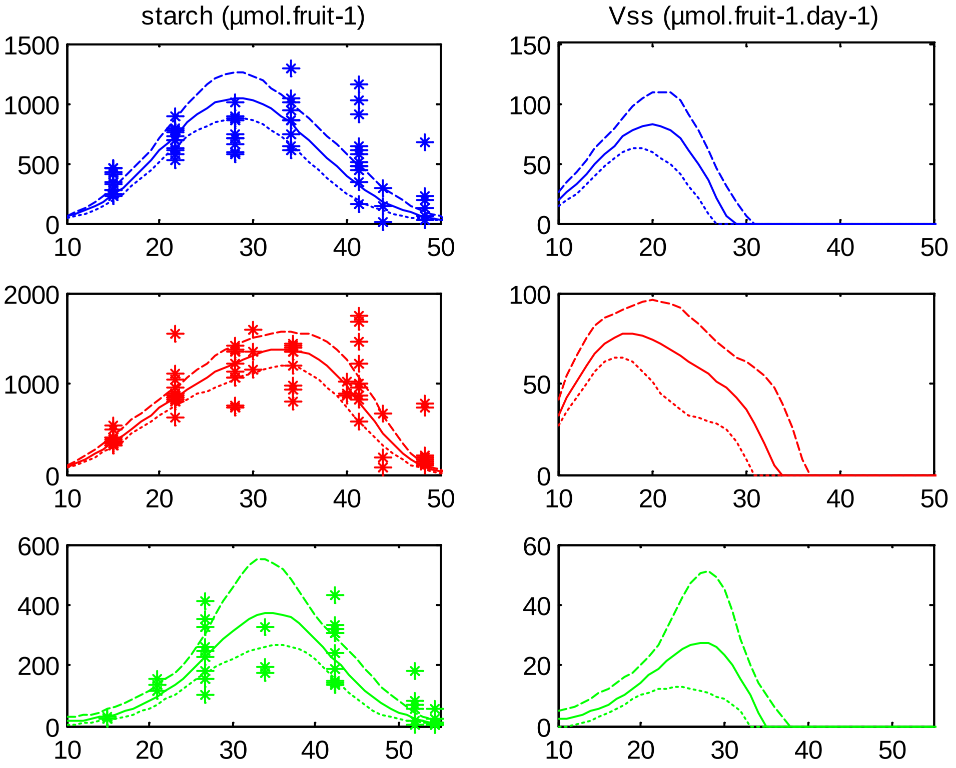
starch (µmol.fruit-1)
Vss (µmol.fruit-1.day-1)
1500
1000
500
0
150
100
50
2000
600
400
200
60
40
20
10
30

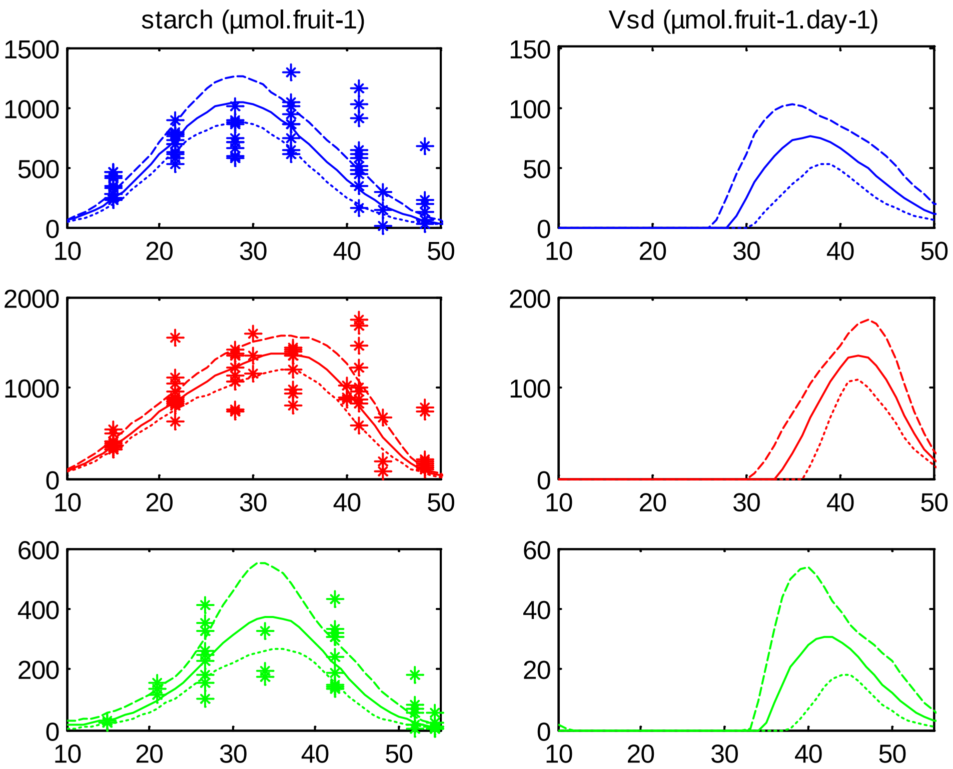
starch (µmol.fruit-1)
Vsd (µmol.fruit-1.day-1)
1500
1000
500
0
150
100
50
2000
200
600
400
60
40
20
10 20 30 40 50

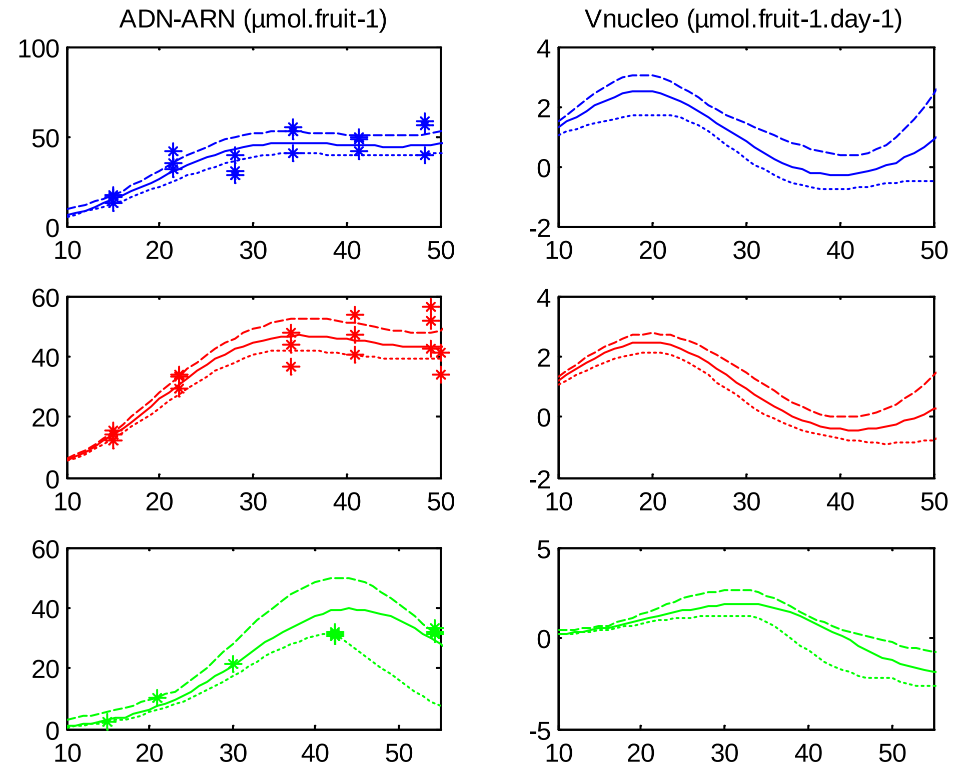
ADN-ARN (µmol.fruit-1)
Vnucleo (µmol.fruit-1.day-1)
100
50
0
4
2
-2
60
40
20
5
-5
10 20 30 40 50

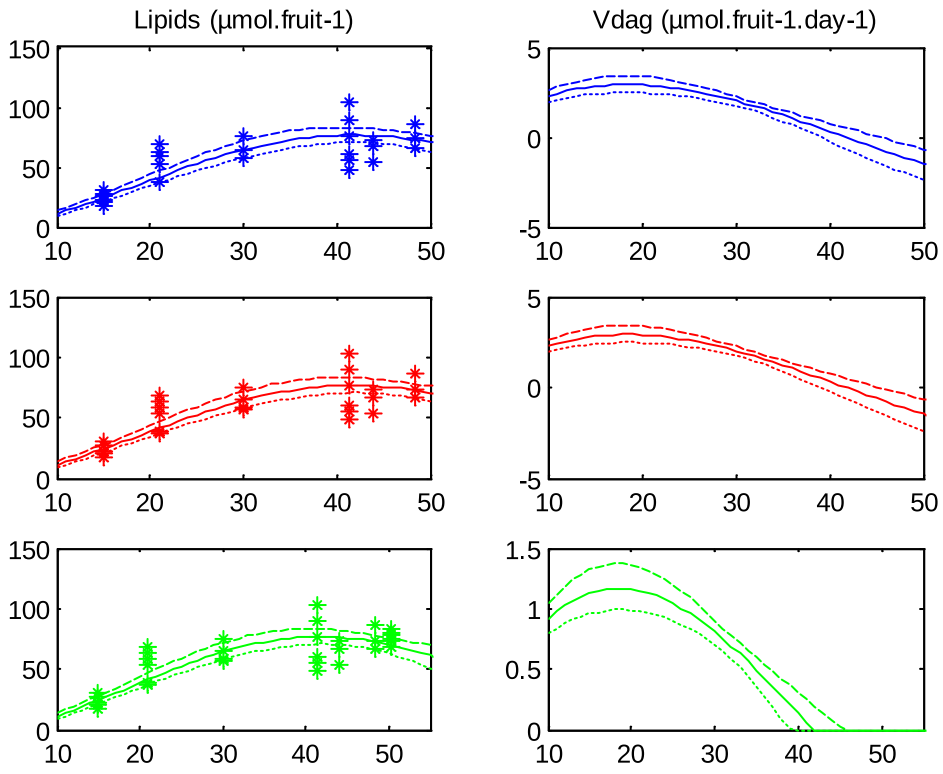

Lipids (µmol.fruit-1)
Vdag (µmol.fruit-1.day-1)
150
100
50
0
10
20
30
40
50
5
0
-5
1.5
1
0.5

Fig. S3 Importance of dissipating fluxes, AOX and UCP, on calculated fluxes. Time-course of (a, b) sucrose uptake, (c, d) CO_2 released, and fluxes involved in tomato fruit energetic metabolism: (e, f) the total ATP flux, (g, h) ATP dissipated in maintenance, (i, j) dissipating fluxes through AOXs and (k, l) UCPs. Fluxes expressed on the fruit-basis (left panel) and on the gFW-basis (right panel) with the model solved on a daily basis with the best fit for outfluxes (black), without UCP (light blue), without AOX (dark blue), and without AOX and UCP (green).

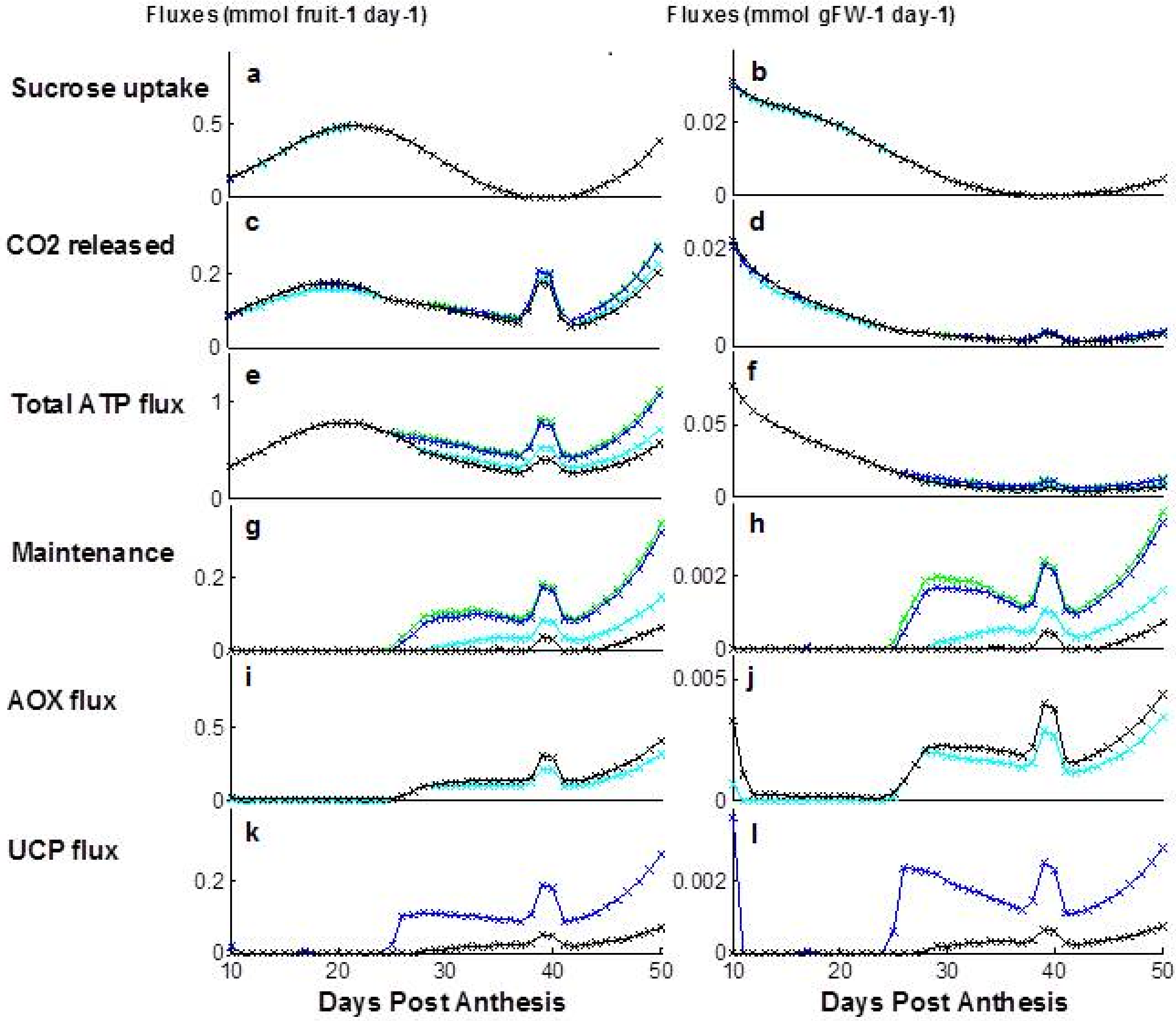

Fig. S4 Plant culture and fruit characterisation. Characterization of tomato cultures under control (black), water stress (red) and shading (green) conditions with (a) electrical conductivity and (b) hygrometry measured into slabs and (c) drainage during the culture. Characterization of fruits with: (d) fruit FW : DW ratios; (e) fruit (closed symbols) and pericarp (open symbols) dry weight contents and (f) carbon and nitrogen contents in (% DW) at several stages (DPA) of development.

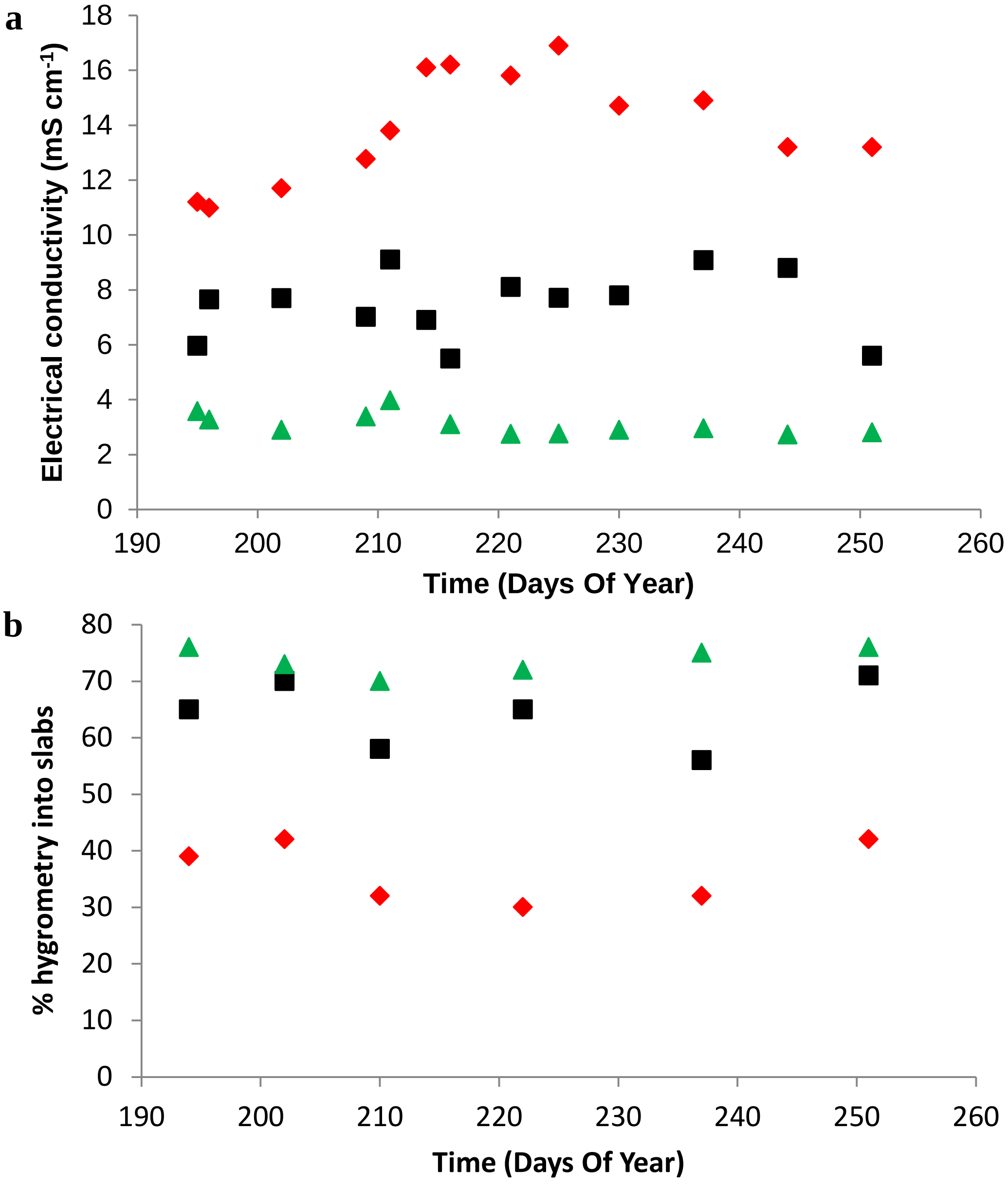
a
Electrical conductivity (mS cm-1)
18
16
14
12
10
8
6
4
2
0
190
200
210
220
230
240
250
260
Time (Days Of Year)
b
% hygrometry into slabs
80
70
60
50
40
30
20
10
0
190
200
210
220
230
240
250
260
Time (Days Of Year)

c

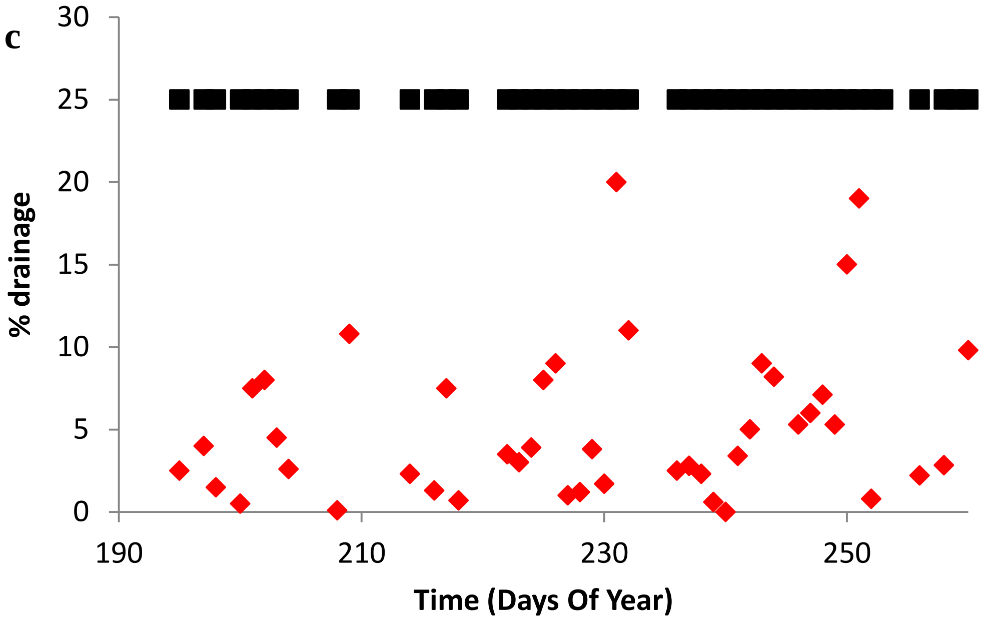

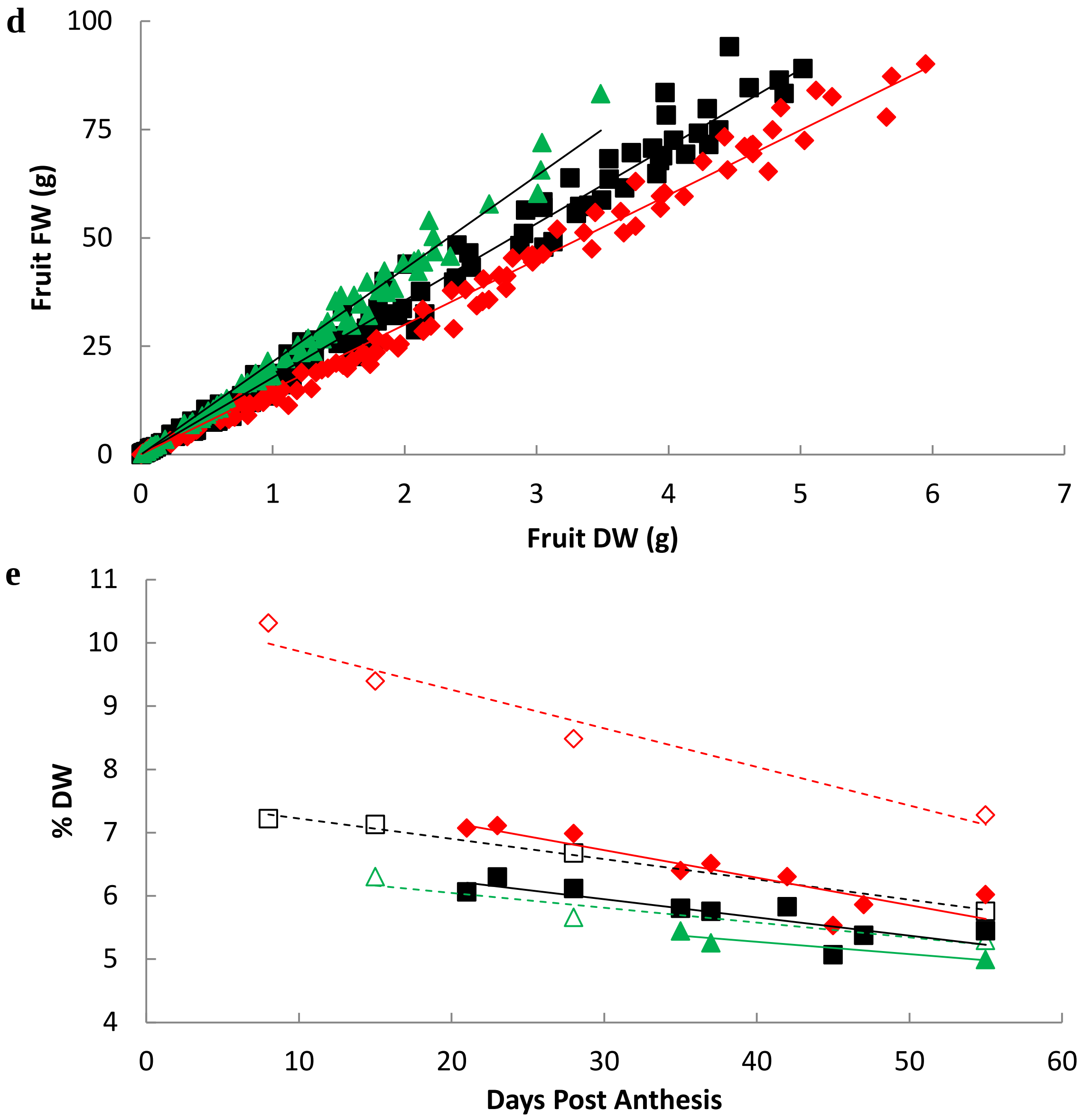
d
100
75
50
25
0
Fruit FW (g)
0
1
2
3
4
5
6
7
Fruit DW (g)
e
11
10
9
8
7
6
5
4
% DW
0
10
20
30
40
50
60
Days Post Anthesis

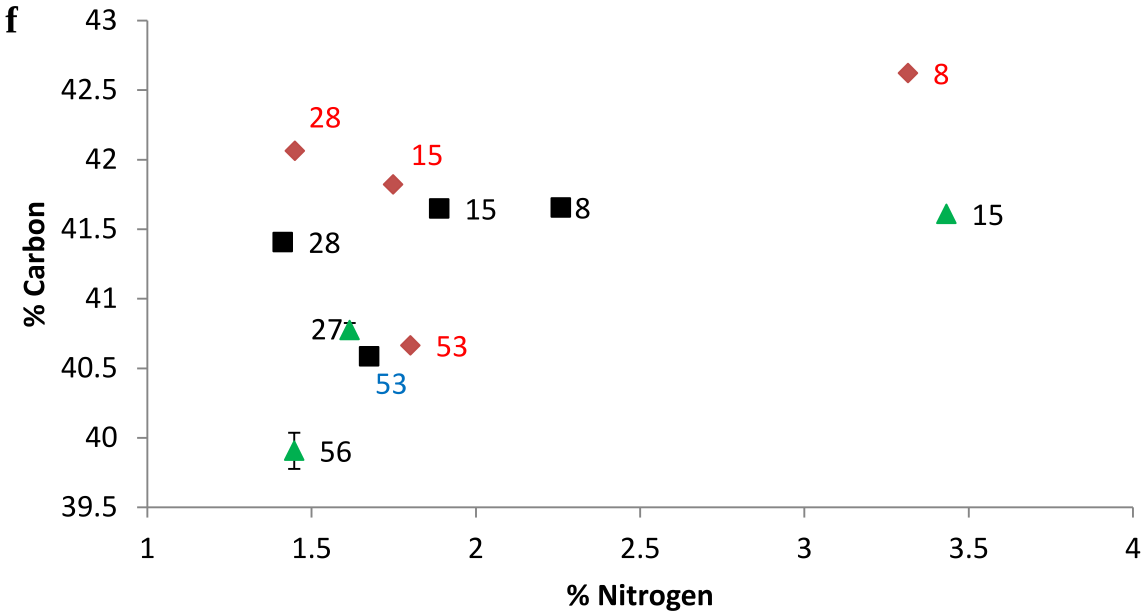
f
43
42.5
42
41.5
41
40.5
40
39.5
% Carbon
1
1.5
2
2.5
3
3.5
4
% Nitrogen
8
28
15
15
8
15
28
27
53
53
56

New Phytologist

Fig. S5 Fruit composition throughout development. Mass balance of tomato fruit pericarp analyzed at nine stages of development under (a) control, (b) water shortage and (c) shading conditions. Accumulated metabolites (sugars, organic acids and free amino acids) and biomass components (starch, cell wall, protein, lipids and nucleic acids) are experimental values expressed in g g^{-1}DW.

a

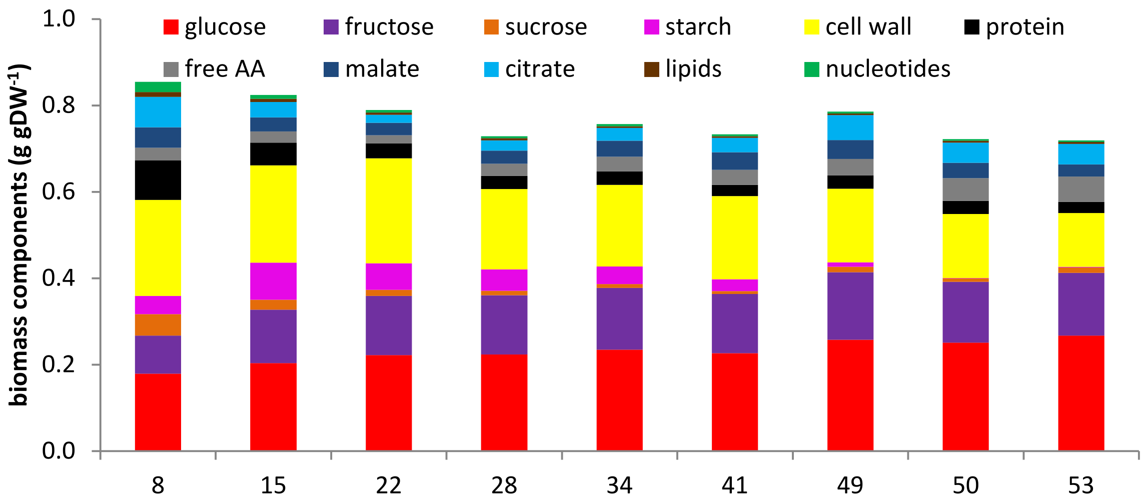

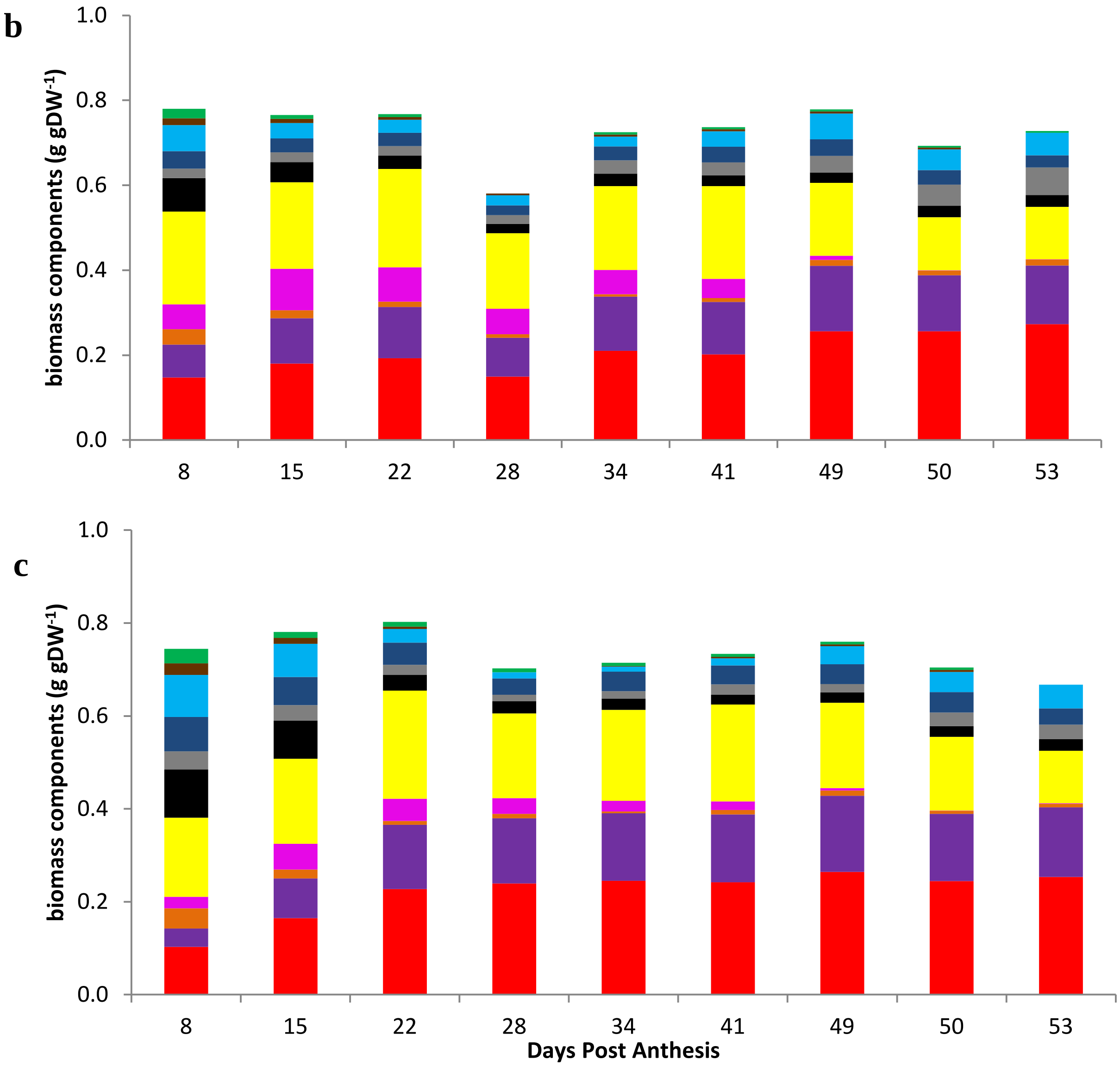
b
c
1.0
0.8
0.6
0.4
0.2
0.0
biomass components (g gDW-1)
8
15
22
28
34
41
49
50
53
Days Post Anthesis

Fig. S6 Origin of CO_2 released throughout tomato fruit development. Percentage of the five calculated fluxes – *Vidh* and *Vkgdh* for TCA, *Vpdh*, *Vme* and *Vg6pdh* – on the total CO_2 released with the model solved on a daily basis using data obtained under (a) control, (b) water stress and (c) shading conditions.

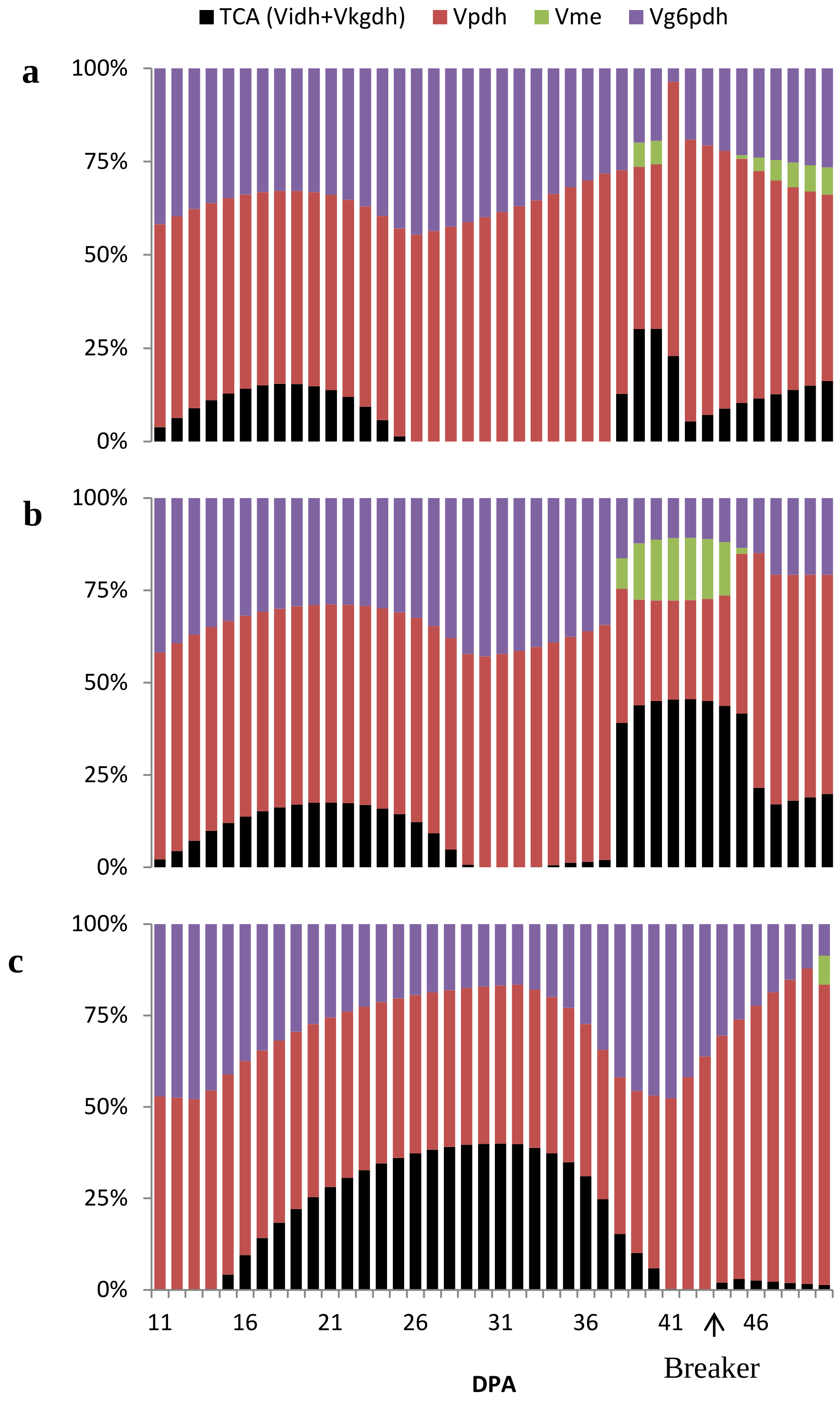

Fig. S7 Galactose and galacturonate throughout the tomato fruit development. Time-course of free galactose (closed symbols) and galacturonate (open symbols) content in tomato fruit pericarp under control (squares), water shortage (triangles) and shading (circles) conditions.

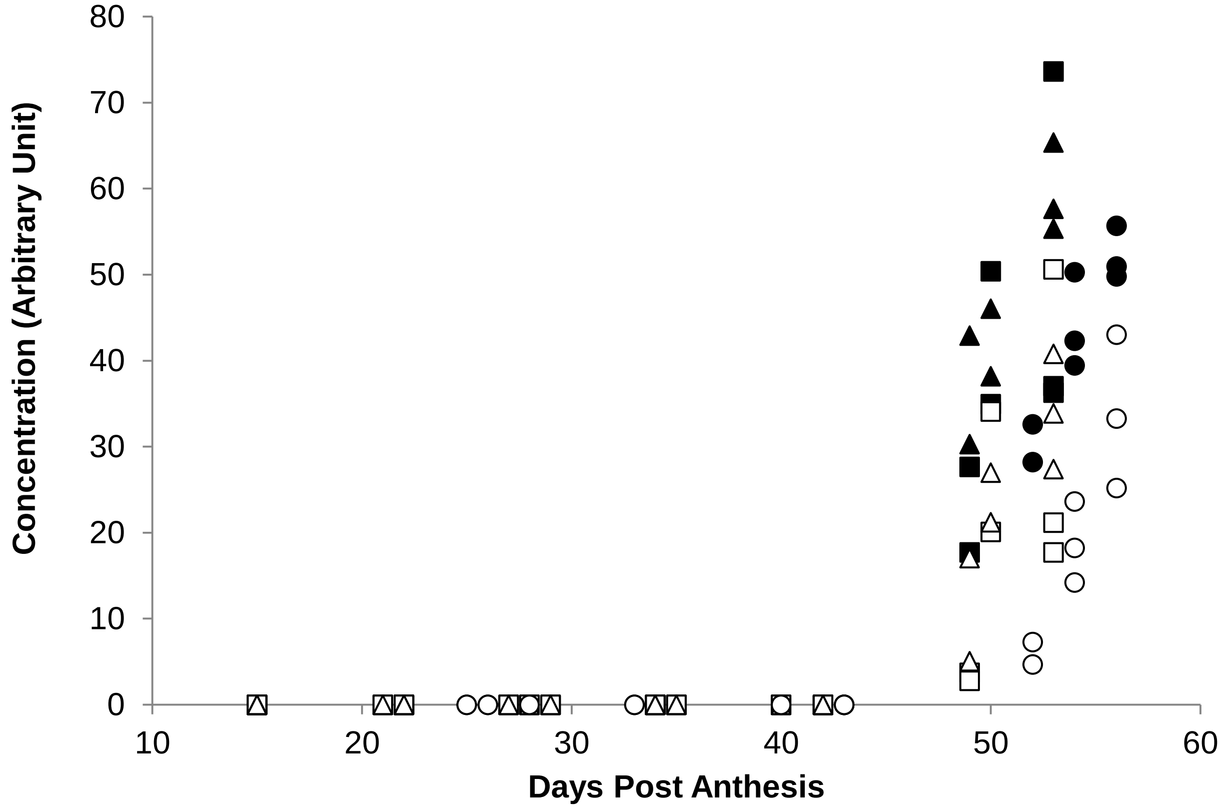

Fig. S8 Estimated heat from metabolic activities. Time-course of the heat production rate estimated from respiration (CO_2 released) with the tomato fruit model solved on a daily basis using data obtained under control (black), water stress (red) and shading (green) conditions.

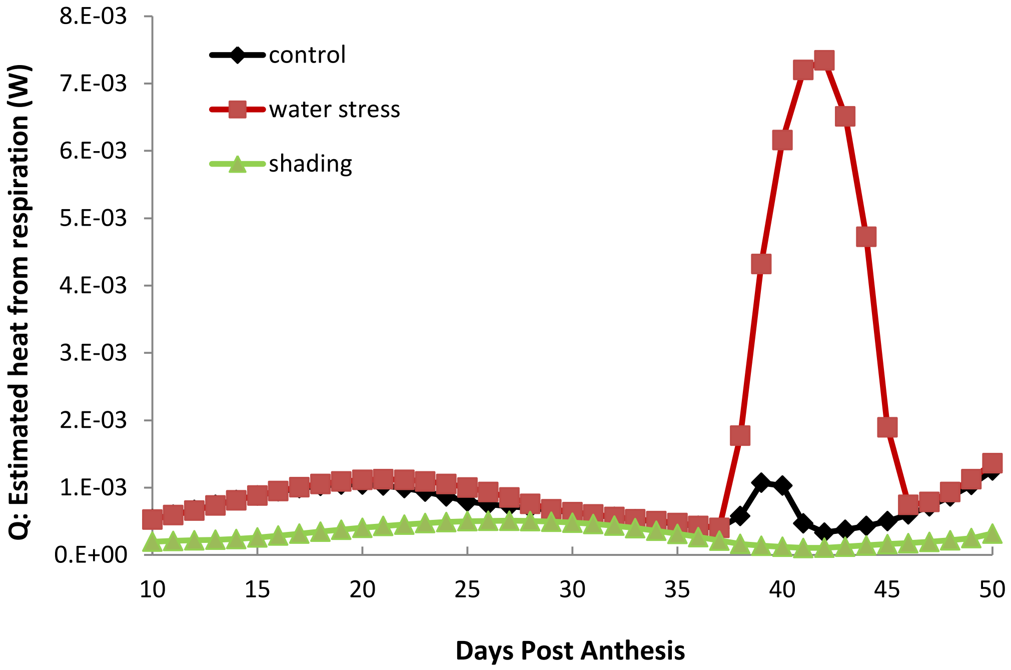

Fig. S9 Fruit temperature and fruit energy variations. (a, e) Fruit age, (b, f) air and median fruit temperatures and (c, g) median fruit energy variations under control (black), water stress (red) and shading (green) conditions and (d, h) a scatter plot of fruit energy variation for the (a, b, c, d) second and the (e, f, g, h) last recording periods.

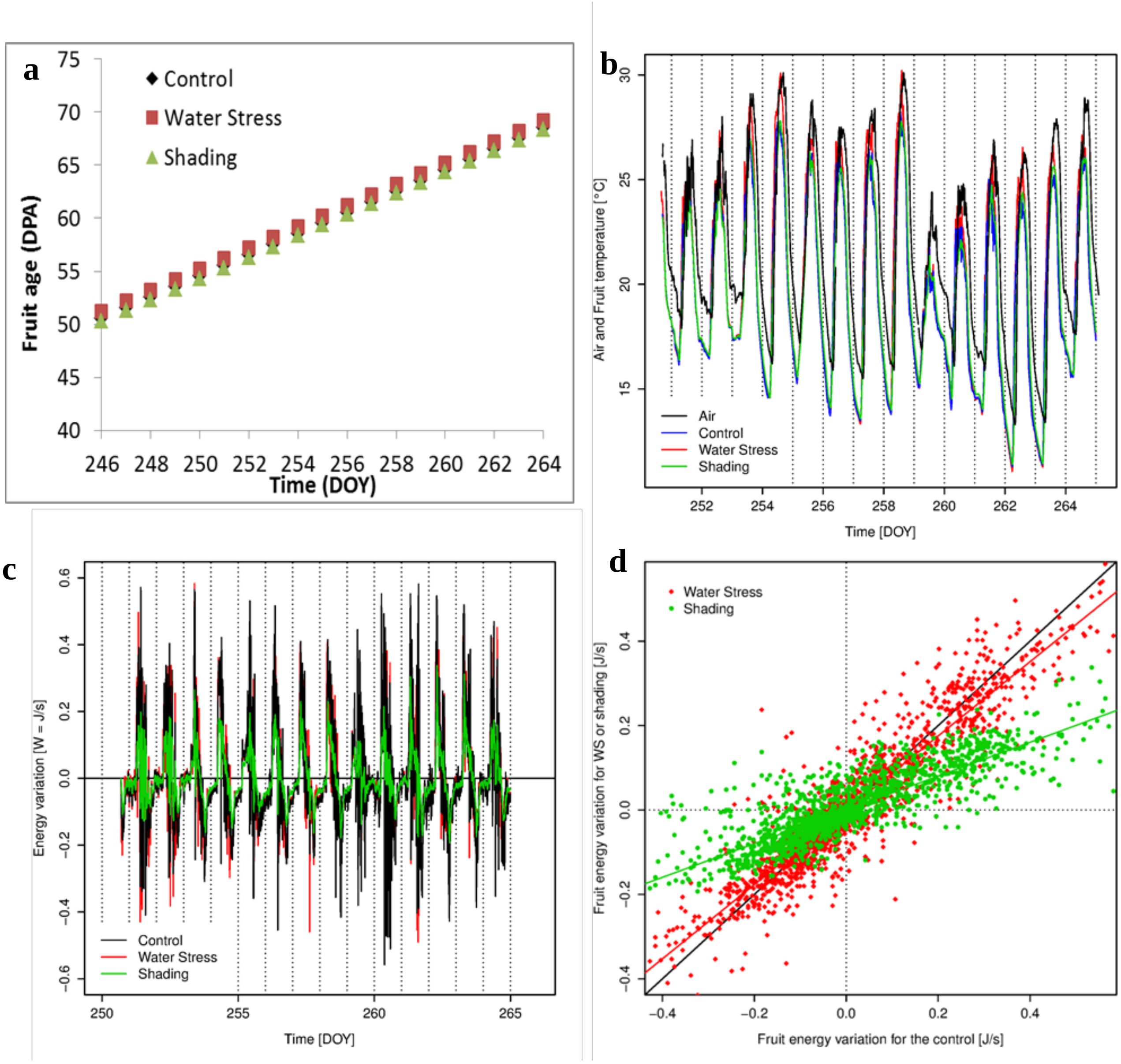

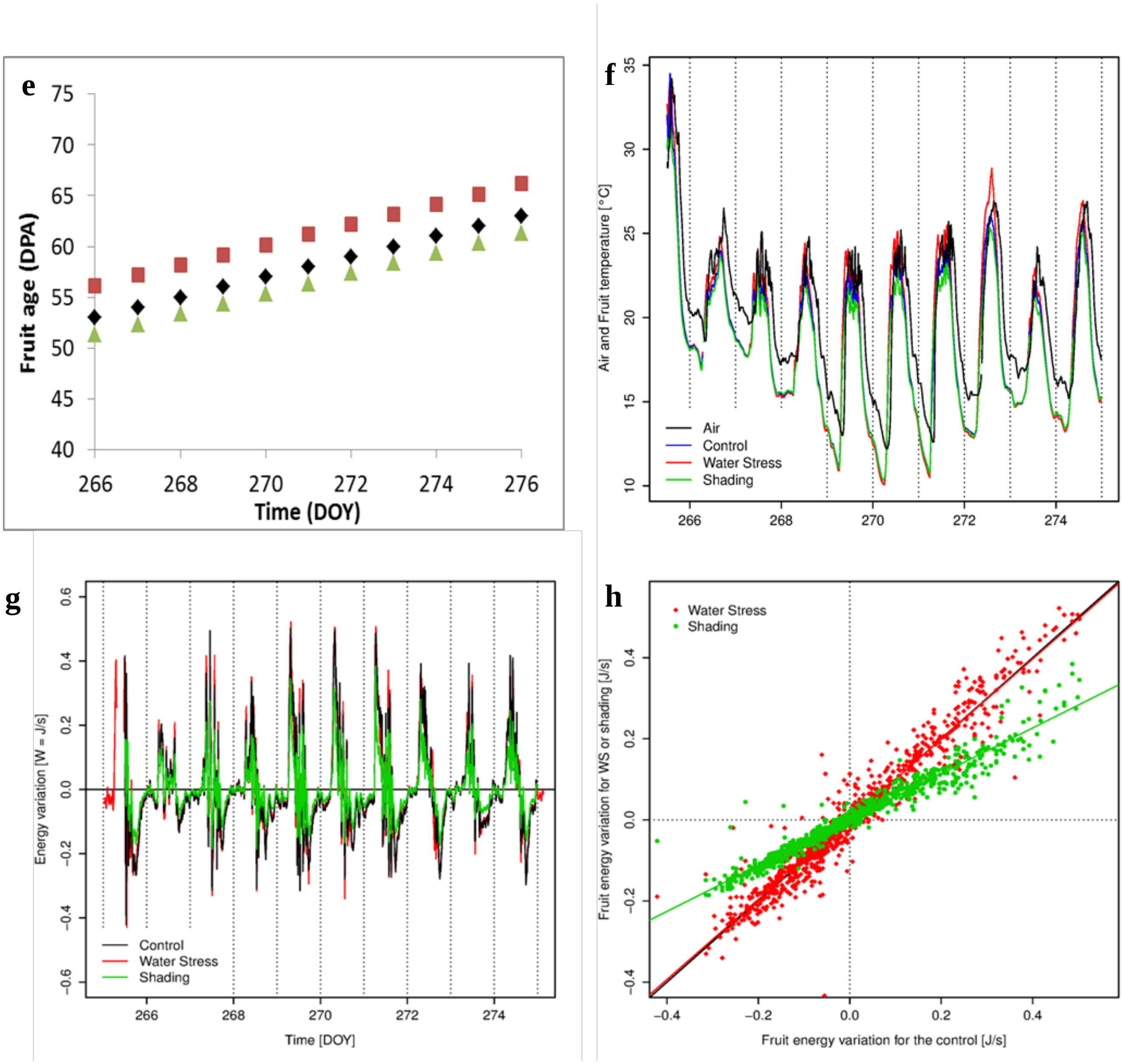
e
Fruit age (DPA)
Time (DOY)
f
Air and Fruit temperature [°C]
Air
Control
Water Stress
Shading
g
Energy variation [W = J/s]
Time [DOY]
Control
Water Stress
Shading
h
Water Stress
Shading
Fruit energy variation for WS or shading [J/s]
Fruit energy variation for the control [J/s]

Video S1 Flux maps of water stress compared to control. Short movie with one map per second comparing fluxes (expressed in mmol fruit^{-1} d^{-1}) calculated solving the model on a daily basis from 2 to 53 DPA for tomato fruit grown under control (on the left) and water stress (on the right) conditions. Flux maps drew with OMIX with red arrows for positive fluxes and blue arrows for negative fluxes, accorded to the convention (Table S1).

Notes S2 Mathematical demonstration of unicity of the flux solution.

Let us recall a standard result on strictly convex functions.

Let f be a continuous strictly convex function on $\mathbb{R}^{n_{int}}$ and A_l a non-empty compact (closed and bounded) subset of $\mathbb{R}^{n_{int}}$.

Theorem 2: The optimization problem of finding some $V_{int}^{*} \in \mathbb{R}^{n_{int}}$ such that

$f(V_{int}^{*}) = min\{f(V_{int}); V_{int} \in A_l\}$ admits a unique solution V_{int}^{*}

Proof: The function f is continuous and is strictly convex that is

$f(\lambda X + (1-\lambda)Y) < \lambda f(X) + (1-\lambda)f(Y)$ for $X \neq Y$ and $\forall\lambda \in]0,1[$

From the Weierstrass extreme values theorem, f continuous from $A_l \subset \mathbb{R}^{n_{int}}$, A_l compact then the minimization problem $minf(X)$ with $X \in A_l$ admits an optimal solution with $X^{*} \in A_l$.

The optimal solution is unique. Indeed if we assume that $X \neq Y$ are two minima of f in A_l

Then we have $f(X) = f(Y) = min\, f$

But as f is strictly convex, $f(\frac{X+Y}{2}) < \frac{f(X)}{2} + \frac{f(Y)}{2} = f(X)$ which leads to a contradiction.

Theorem 1: The problem

$Minimize\ f(V) = \sum_{i=1}^{n}(v_i)^2$ (3)

Subject to the constraints:

$$NV = 0$$

$$V_{int,min}^{l} \leq V_{int} \leq V_{int,max}^{l} \quad \text{and} \quad V_{ext} = V_{ext}^{l}$$

admits a unique solution.

<u>**Proof**</u>: The stoichiometry matrix N can be reordered to be partitioned into 1×2 blocks $N = (N_1 \quad N_2)$

where N_1 is a m_{int}xn_{int} matrix, N_2 is a m_{int}xn_{ext} matrix so that the steady state constraints $NV = 0$ can be rewritten by $\begin{pmatrix} \frac{dX_{I,int}}{dt} \\ \frac{dX_{E,int}}{dt} \end{pmatrix} = (N_1 \quad N_2)\begin{pmatrix} V_{int} \\ V_{ext} \end{pmatrix} = 0 \Leftrightarrow N_1 V_{int} = -N_2 V_{ext}$.

Then problem (3) is the optimization problem of finding some $V_{int}^* \in \mathbb{R}^{n_{int}}$ such that

$$f(V_{int}^*) = min\{f(V_{int}); V_{int} \in A_l\}$$

Where, for l=1,…,50 (corresponding to the stages of fruit development),

$$A_l = \left\{V_{int} \in \mathbb{R}^{n_{int}}; N_1 V_{int} = -N_2 V_{ext}^l; V_{int,min}^l \leq V_{int} \leq V_{int,max}^l;\right\} \subset \mathbb{R}^{n_{int}}$$

is the feasible set and $f: \mathbb{R}^{n_{int}} \to \mathbb{R}$ is the objective function.

As A_l is a compact convex subset of $\mathbb{R}^{n_{int}}$ (see lemma 1 below) and f is a strictly convex and continuous function on $\mathbb{R}^{n_{int}}$, from theorem 2, this strictly convex problem admits a unique solution V_{int}^* (for each $l = 1...50$).

Lemma 1: A_l is a compact convex subset of $\mathbb{R}^{n_{int}}$

Proof : set

$$A_{1_l} = \left\{V_{int} \in \mathbb{R}^{n_{int}}; \ V_{int,min}^l \leq V_{int} \leq V_{int,max}^l\right\}$$

$$A_{2_l} = \{V_{int} \in \mathbb{R}^{n_{int}}; \ N_1 V_{int} = -N_2 V_{ext}^l\}$$

1. A_l is bounded because $A_l \subset A_{1l}$ which is bounded.
2. Set $b = -N_2 V_{ext,l}$. We check numerically that $rank(N_1 \,|b) = rank(N_1)$. So according to the Rouché–Capelli theorem, the linear system $N_1 V_{int} = b$ admits solutions. The subset A_{2_l} is not empty and is closed. Then A_l is closed as intersection of two closed subsets A_{i_l}.
3. For $i \in \{1,2\}, \forall (X, Y) \in A_{il}$, it is easy to prove that $\forall \lambda \in [0,1]$ we also have $\lambda X + (1 - \lambda)Y \in A_{il}$. A_l is convex as intersection of two convex subsets.

So A_l is a compact convex subset.
